# cGAS-STING pathway in innate immunity and its cell-specific role in kidney diseases

**DOI:** 10.3389/fimmu.2026.1763106

**Published:** 2026-06-23

**Authors:** Miaotao Wei, Huasheng Luo, Wanglong Liu, Tongtong Ma, Peng Wang

**Affiliations:** 1 Guangdong Provincial Key Laboratory of Autophagy and Major Chronic Non-Communicable Diseases, Institute of Nephrology, Affiliated Hospital of Guangdong Medical University, Zhanjiang, Guangdong, China; 2 Key Laboratory of Prevention and Management of Chronic Kidney Diseases, Institute of Nephrology, Affiliated Hospital of Guangdong Medical University, Zhanjiang, Guangdong, China; 3 The First Clinical College, Guangdong Medical University, Zhanjiang, Guangdong, China; 4 Department of Anesthesiology, Affiliated Hospital of Guangdong Medical University, Zhanjiang, Guangdong, China

**Keywords:** cGAS-STING, innate immunity, kidney disease, therapeutic targets, tubular epithelial cell

## Abstract

The cyclic GMP-AMP synthase (cGAS)-stimulator of interferon genes (STING) signaling pathway is a key component of the innate immune system, responding to the presence of DNA within cells to trigger an inflammatory response. A growing body of research shows that this pathway is equally important in non-infectious diseases such as cancer, metabolic diseases, and autoimmune diseases. In the kidney, the cGAS-STING signaling pathway is activated in different types of renal cells and can drive a range of disease-causing processes, including inflammation, fibrosis, and functional decline. In this review, we systematically summarize the basic mechanism of the cGAS-STING pathway and its role in innate immunity. In particular, we discuss the specific effects of this pathway in renal cells and discuss its potential applications in future basic research and clinical treatment, providing a theoretical basis for the development of new therapies for kidney diseases.

## Introduction

The cyclic GMP-AMP synthase (cGAS)-stimulator of interferon genes (STING) pathway is a core component of the innate immune system, primarily responsible for detecting cytoplasmic DNA and initiating immune responses (1). After sensing DNA, cGAS synthesizes the second messenger cyclic GMP-AMP (cGAMP), activating STING (2). This activation triggers a downstream signaling cascade, including the production of type I interferon (IFN-I) and pro-inflammatory cytokines (3). Although this response is critical for host defense, dysregulated activation of STING has been implicated in the pathogenesis of numerous inflammatory, autoimmune, and metabolic disorders (4, 5). It is worth noting that dysregulation of the cGAS-STING pathway is related to the pathogenesis of kidney disease, and its activation drives inflammation, fibrosis, and cell damage, promoting disease progression (6, 7).

Kidney disease is a significant global health burden, with chronic kidney disease (CKD) affecting more than 10% of the global population and acute kidney injury (AKI) being a major risk factor for the progression of CKD (8, 9). Activation of the cGAS-STING pathway has been shown to play an important role in various renal cell types such as podocytes, tubular epithelial cells (TECs), immune cells and vascular smooth muscle cells, driving pathological processes such as inflammation, fibrosis, and cell injury (10–13). The role of the cGAS-STING pathway in different renal cells underscores its importance in the pathogenesis of kidney disease and highlights its potential as a therapeutic target (14, 15). Although the pathologic role of the cGAS-STING pathway in renal diseases has been initially revealed, its diversity and specific mechanisms in different renal cell types remain to be further studied.

In this review, we systematically review the basic mechanism of the cGAS-STING pathway and its role in innate immunity, with a particular focus on its cell-specific effects in kidney diseases. At the same time, we summarize the current status of basic research and clinical treatment applications of this pathway in kidney diseases, and aim to facilitate the transition of cGAS-STING targeted therapy from the basic research stage to the clinical application stage, providing innovative treatments for kidney diseases.

## The role of the cGAS-STING pathway in innate immunity

### The core activation mechanism of the cGAS-STING signaling pathway

**Figure 1:**
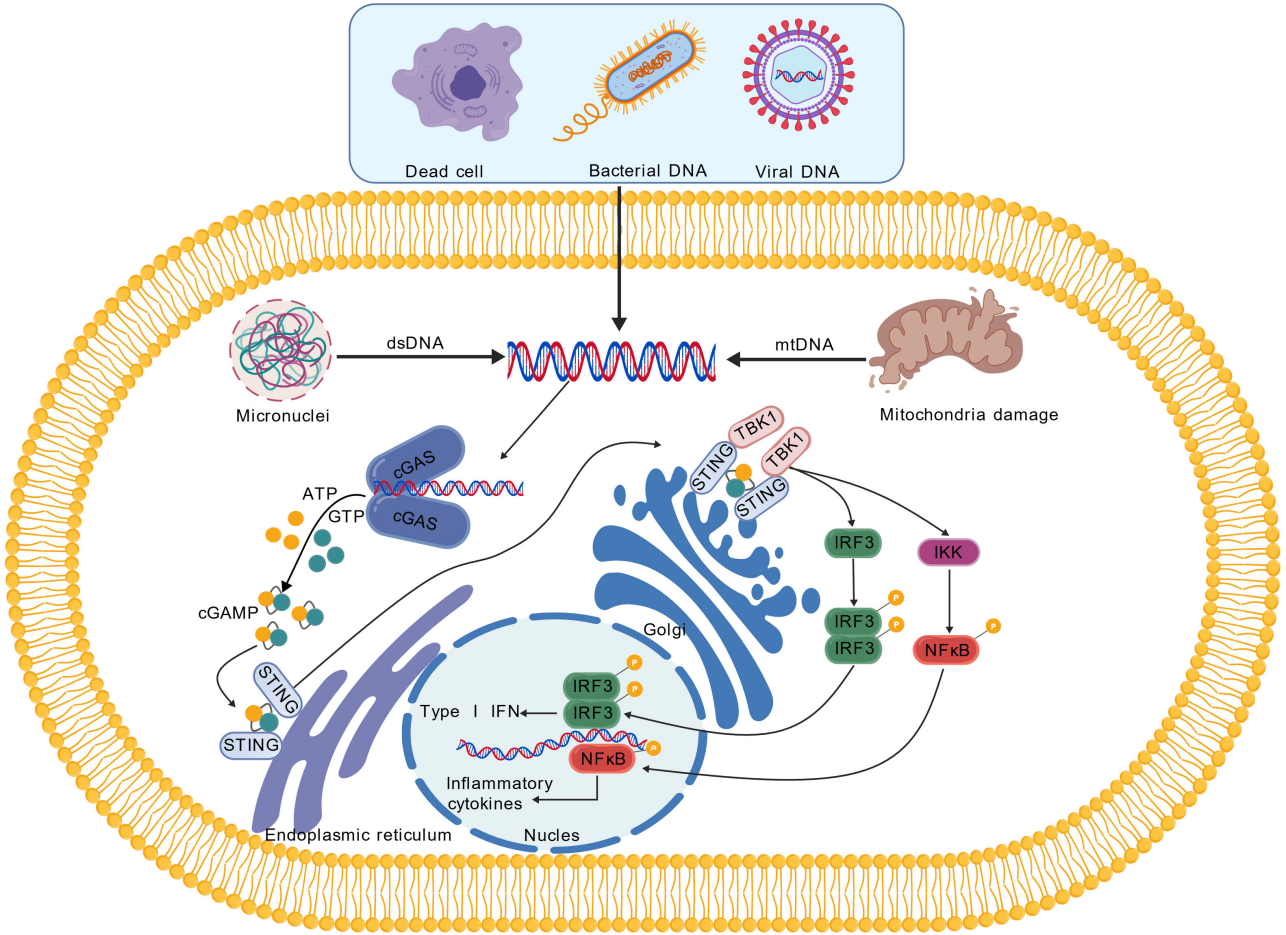
Composition and downstream cascade reaction of cGAS-STING signaling pathway. cGAS recognizes DNA from dead cells, bacteria, viruses, micronucleus, and damaged mitochondria. After detection of DNA, cGAS forms a dimer, which catalyzes the synthesis of cGAMP using ATP and GTP as substrates. cGAMP acts as a second messenger to bind and activate the adaptor protein STING on the ER. Activated STING is transported to the Golgi, forms aggregates on the Golgi membrane, and recruits and activates TBK1. TBK1 phosphorylates the transcription factor IRF3, causing it to form a dimer that is incorporated into the nucleus and thus initiates IFN-I expression. In addition, STING activates IKK, releases NF-κB into the nucleus, and induces the production of TNF-α, IL-6 and other proinflammatory factors. Created with BioGDP.com (31).

cGAS is a well-established cytoplasmic DNA sensor whose main function is to identify DNA molecules that are abnormal in the cytoplasm, including pathogen DNA or DNA leaked by the host itself, and this recognition ability makes it a core molecule for monitoring the danger signals of the immune system (16–18). It is noteworthy that cGAS has also been discovered to localize in the nucleus. Nevertheless, within the nucleus, the interaction of cGAS with the cell’s endogenous genomic DNA is precisely regulated by nucleosomes and nuclear protein interactions, which effectively prevents abnormal activation against self-DNA. It only reacts under circumstances of severe chromatin damage, such as DNA damage and micronucleus formation (19–24). Upon detection of DNA by cGAS, the enzyme undergoes dimerization and uses ATP and GTP as substrates to catalyze the synthesis of the cyclic dinucleotide (CDN), cyclic GMP-AMP (2’,3’-cGAMP) (25). As a second messenger, cGAMP binds to and activates the adaptor protein STING on the endoplasmic reticulum. The activated STING is transported to the Golgi apparatus via vesicles, where it forms aggregates on the Golgi membrane and recruits and activates TANK-binding kinase 1 (TBK1) (26, 27). TBK1 phosphorylates the transcription factor IRF3, causing it to form a dimer incorporated into the nucleus that initiates the expression of IFN-I and interferon-stimulated genes. In addition, STING activates IκB kinase through the adaptor protein TRAF6, leading to the degradation of IκBα and the release of NF-κB into the nucleus to induce the production of pro-inflammatory factors such as TNF-α and IL-6 (28–30). In summary, the cGAS-STING pathway, through the detection of abnormal cytoplasmic DNA, promptly initiates a potent IFN-I and inflammatory response, thereby establishing a core defense line for anti-infection immunity. Meanwhile, its precise inhibitory mechanism for nuclear activity guarantees that this system can accurately differentiate between “self” and “non-self” DNA, and enables it to monitor genomic stability and respond to endogenous danger signals (see **Figure 1**).

### The cGAS-STING signaling pathway is a critical mechanism for host defense against pathogen invasion

Bacterial DNA, acting as a danger signal, is detected by cGAS in the cytoplasm, which subsequently activates STING. This activation triggers a cascade of downstream signaling events that induce the production of IFN-I and inflammatory factors (32–34). In bone marrow-derived macrophages (BMDMs) from cGAS- or STING-deficient mice, the production of IFN-I and cytokines is significantly reduced following bacterial infection (35–37). The number of bacteria known to activate the cGAS-STING pathway is increasing and includes species such as Propionibacterium acnes, Mycobacterium, Listeria, Staphylococcus aureus, Francisella, Salmonella, Streptococcus, Legionella pneumophila, and Chlamydia (35–49). In the absence of cGAS, the induction of IFN-I and cytokines by most bacteria is essentially abolished. Additionally, CDNs secreted by Listeria and Chlamydia, such as c-di-AMP and c-di-GMP, serve as diffusible signaling molecules that can directly cross the cell membrane or enter the cytoplasm via transporters to bind and activate STING (50). This mechanism may represent an equally important or even more critical mode of activation in certain infection contexts, ensuring that bacteria can trigger or modulate the host immune response even when evading DNA recognition (51, 52).

Currently, reports on the activation of the cGAS-STING pathway by DNA viruses are increasing (53–59). In mice lacking cGAS or STING, no IFN-β is produced during infection with HSV-1, murine γ-herpesvirus 68, or VACV, resulting in increased viral titers. Additionally, mice deficient in cGAS and STING are more susceptible to RNA virus infections (60). cGAS also serves as a key receptor for retroviruses. It can induce an interferon response by detecting intermediate cDNA products generated during HIV reverse transcription, thereby inhibiting the activation of the virus’s latent reservoir. The expression level of cGAS in peripheral blood mononuclear cells of HIV-infected individuals correlates with viral control. During this process, some cDNA is directly transferred to the nucleus and integrated into the host genome (61–63). Consequently, retroviruses typically do not trigger a strong innate immune response.

The release of endogenous danger signals, such as mitochondrial DNA (mtDNA) and damage-associated molecular patterns (DAMPs), during pathogen invasion is a crucial mechanism for amplifying and sustaining the activation of the cGAS-dependent pathway, thereby linking infection to sterile inflammation. The production of IFN-I coordinates the immune response, eliminates pathogens, and limits tissue damage. However, continuous overactivation of IFN-I and inflammatory factors can disrupt immune homeostasis, directly causing cell death or impairing essential antibacterial immunity, which exacerbates disease severity (64, 65). This dual role of protection and harm remains an important area for further research (see **Table 1**).

**Table 1:** The cGAS-STING signaling pathway defends against pathogen invasion.

Pathogen category	Signaling transduction process	Functional impact	Refs
Cutibacterium acnes	Activates the cGAS-STING pathway and induces the formation of the IFN-I signaling axis	Inflammatory cytokines↑	(38)
Mycobacterium tuberculosis	Mtb DNA binds to cGAS to stimulate the production of cGAMP	Type-I-IFN and IL-1β↑;Autophagy↑	(35, 39, 40)
Listeria monocytogenes	Listeria DNA binds to IFI16/cGAS -STING to induce the IFN response	IFN-β↑	(36)
	TBK1 phosphorylates and activates MVB12b, and separates the DNA of Listeria into extracellular vesicles	T cell proliferation↓;Apoptosis↑	(41)
Staphylococcus aureus	Activates the STING/IRF 3 pathway	IFN-β↑	(37)
Francisella novicida	F. novicida dsDNA binds to cGAS	Type-I-IFN↑	(42)
	cGAS and Ifi204 cooperates to sense F. novicida dsDNA and activates STING	Type-I-IFN↑	(43)
Salmonella	mtDNA release activates the cGAS-STING pathway	Type-I-IFN↑	(44)
	Overexpression of cGAS to produce cGAMP activates STING	Human macrophages and dendritic cells type-I-IFN↑	(45)
Group B Streptococcus	GBS expresses exonuclease, which hydrolyzes cyclic-di-AMP, resulting in reduced STING activation	Type-I-IFN↓	(46)
Streptococcus pneumoniae	Monocytes produces IL-12p70, which affects the cGAS-STING and MyD88 pathways	Late-stage IFNγ↑	(47)
Legionella pneumophila	HAQ STING variant impairs cGAS-dependent antibacterial responses	Type-I-IFN↓	(48)
Chlamydia psittaci	Induces mitochondrial oxidative stress and damage to activate the cGAS-STING-IRF 3/NLRP 3 pathway	Type-I-IFN and Il-1β↑	(49)
Cytomegalovirus	cGAS senses cytosolic viral DNA and catalyzes cGAMP production	Type-I-IFN↑	(53)
	Early activation of the cGAS-STING-IRF 3 pathway	Type-I-IFN↑	(54)
Kaposi sarcoma herpesvirus	Latently associated nuclear antigen of the cytoplasmic isoform binds directly to cGAS and inhibits phosphorylation of TBK 1 and IRF 3	Type-I-IFN↓; KSHV cleavage replication↑	(55)
	γ-herpesvirus-specific tegument protein (KSHV ORF52) directly inhibit cGAS enzymatic activity	Type-I-IFN↓	(56)
	Viral interferon regulatory factor 1 prevents STING from interacting with TBK 1	IFN-β↓	(57)
HSV-1	cGas (-/-) mice were lethally infected	Type-I-IFN↓	(58)
HIV	HIV reverse transcription activates cGAS to produce cGAMP	Type-I-IFN↑	(61)
	CD 4+ T cells sense HIV-1 infection by regulating cGAS via viral accessory proteins Vpr and Vpu	Type-I-IFN↑	(62)
	NONO protein binds to the HIV-2 capsid and facilitates cGAS binding to DNA	Type-I-IFN↑	(63)

↑ indicates an increase or elevation in the expression level compared to the control group or baseline; ↓ indicates a decrease or reduction.

### The cGAS-STING signaling pathway in diseases

Dysregulated or chronic activation of cGAS-STING pathway may cause immunopathological damage, leading to autoimmune diseases, inflammatory diseases, tumors, aging, metabolic diseases and organ-specific diseases (66). It has been reported that cGAS is distributed in small amounts in the nucleus but is bound by histone H2A-H2B heterodimer in nucleosomes, inhibiting its interaction with nuclear DNA (20, 22, 24). Chromatin deaggregation leads to the destruction of the binding of histone H2A-H2B to cGAS. After the autoantibody-nucleosome complex is ingested by phagocytes, nuclear DNA leaks into the cytoplasm, abnormally activates the cGAS-STING signaling pathway, and continues to produce IFN-I, which drives the occurrence of autoimmune diseases such as systemic lupus erythematosus (21, 23, 67). The TREX1 gene is responsible for the degradation of cytoplasmic DNA. Loss-of-function mutations in TREX1 lead to its own DNA accumulation, which continuously activates the cGAS-STING pathway and causes severe encephalitis and skin lesions (68–70). Genetic studies have shown that STING gain-of-function mutations can lead to early-onset systemic inflammation (71).

**Figure 2:**
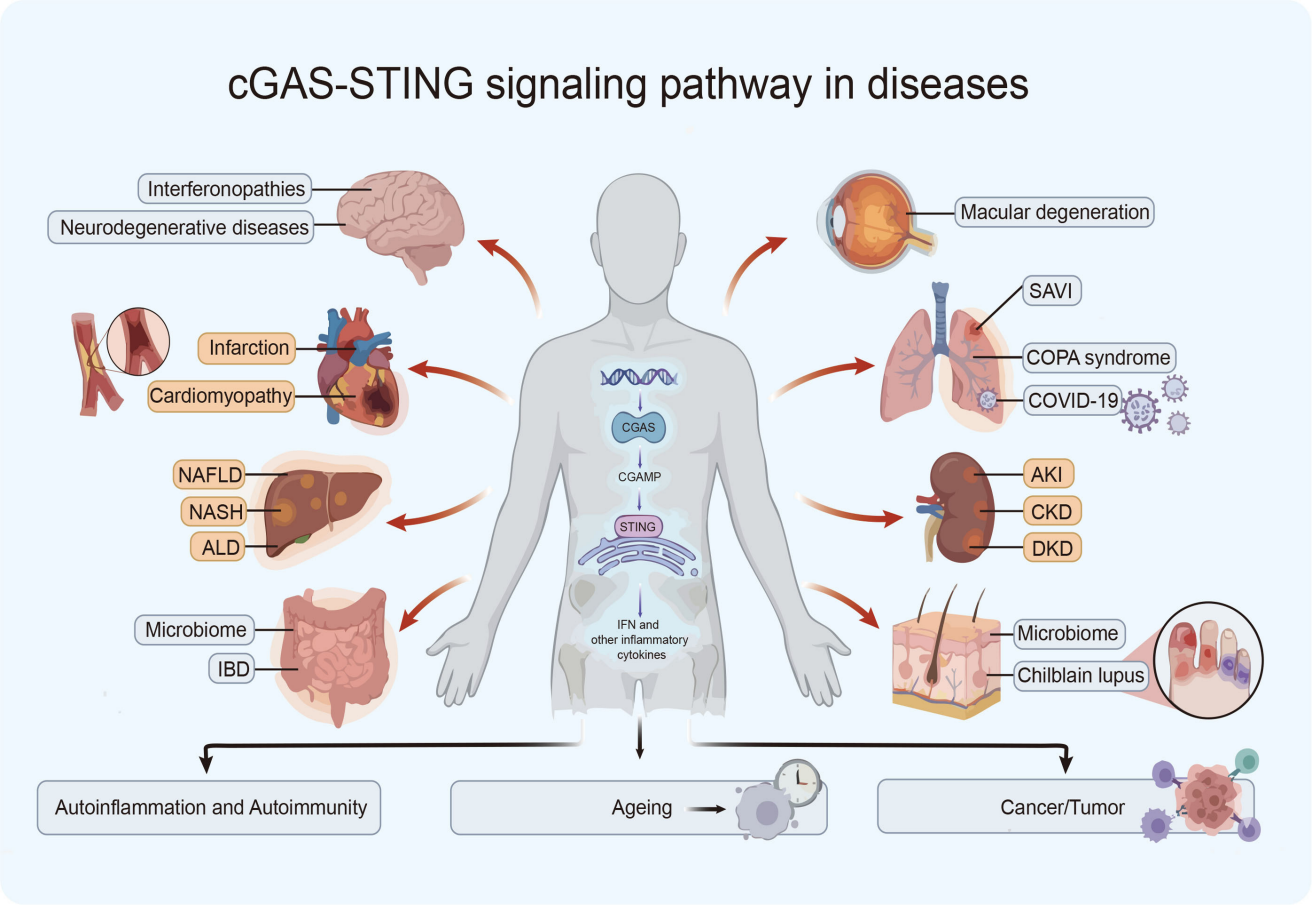
The cGAS-STING signaling pathway in diseases. The cGAS-STING signaling pathway is involved in various systemic disease processes, including systemic inflammatory diseases, aging and cancer, as well as specific diseases affecting organs such as the eyes and the brain. AKI, acute kidney injury; CKD, chronic kidney disease; DKD, diabetic kidney disease; COPA, coatomer protein subunit-α; IBD, inflammatory bowel disease; NAFLD, non-alcoholic fatty liver disease; NASH, non-alcoholic steatohepatitis; ALD, alcoholic liver disease; SAVI, STING-associated vasculopathy with onset in infancy. Figure created with BioRender.com.

In addition to its important role in autoimmune diseases, the cGAS-STING pathway, as a key component of the innate immune system, also plays a central role in regulating inflammatory responses. Inflammatory diseases are often accompanied by cell damage and tissue destruction, and the released DNA fragments may activate the immune response through the cGAS-STING pathway, further amplifying the inflammatory cascade (72). The cGAS-STING pathway plays an important role in inflammation in a variety of organs, which is not only involved in defense against pathogen infection, but also may lead to pathological inflammation and organ damage. In recent years, there have been many reports on lung diseases (73), liver diseases (74), kidney diseases (75), heart diseases (76), gastrointestinal diseases and joint inflammatory diseases (see **Figure 2**) (77, 78).

Chromosome instability of tumor cells leads to nuclear DNA leakage into the cytoplasm via micronuclei, which activates STING to promote inflammation in the premetastatic microenvironment and accelerate metastasis (79). STING up-regulates TGF-β through NF-κB, promotes tumor matrix fibrosis, and inhibits T cell infiltration. DNA damage in tumor cells induced by radiotherapy or chemotherapy releases a large number of chromosomal fragments, activates the cGAS-STING pathway, and promotes dendritic cell maturation and T cell infiltration (80). However, certain tumors also downregulate cGAS expression by promoter methylation or secrete exosomes carrying DNase to evade immune recognition (81). Cytosolic DNA accumulated in senescent cells triggers senescence associated secretory phenotype through cGAS-STING to promote tissue degradation (79). In addition, mtDNA leakage activated the cGAS-STING pathway and induced insulin resistance in adipose tissue macrophages of obese mice (82). STING activation in adipose tissue macrophages promotes the secretion of IL-6 and TNF-α, inhibits insulin signaling pathway, and aggravates metabolic disorders (83). Mitochondrial damage in hepatocytes releases mtDNA and activates the cGAS-STING pathway to drive liver inflammation and fibrosis, leading to the occurrence of non-alcoholic steatohepatitis (NASH). STING-deficient mice have reduced liver steatosis after high-fat diet (84). Ubiquitin-specific protease 18 (USP18) directly binds to STING, deubiquitinates it, and promotes lipopolysaccharide (LPS)-induced ferroptosis in human renal organoids (85). Additionally, palmitoylation of STING at cysteine residues C88 and C91 facilitates its interaction with the mitochondrial voltage-dependent anion channel VDAC2, thereby preserving mitochondrial function and promoting the progression of renal cell carcinoma (RCC) (86).

The activity and function of the cGAS-STING pathway are also regulated by a variety of post-translational modification mechanisms, including phosphorylation, acetylation, ubiquitination, deubiquitylation, glutamylation, sumoylation, palmitoylation, and N-glycosylation. For details, please refer to the recently published relevant reviews (87, 88).

## The cGAS-STING signaling pathway in renal cells

The kidney is a complex and highly vascularized organ, which is crucial for maintaining body homeostasis. The kidneys filter blood to remove metabolic waste products such as urea, ammonia, and bile by-products from the blood and ultimately form urine, while regulating the pH of water, electrolytes, and tissue fluid (89). In addition, the kidney regulates blood pressure through the renin-angiotensin-aldosterone system, secrete erythropoietin to stimulate erythropoiesis, and participate in the regulation of vitamin D activation to regulate the balance of calcium and phosphorus (90). As a highly metabolically active organ rich in mitochondria, kidney function depends on continuous energy supply and precise cellular regulation. However, this property also makes the kidney vulnerable to various injuries, such as ischemia-reperfusion, sepsis and nephrotoxins, resulting in cellular stress and dysfunction (91). During these injuries, cells in the kidney, such as TECs, podocytes, endothelial cells, mesangial cells, renal interstitial fibroblasts, and immune cells, are important targets due to their critical roles in maintaining renal functions such as filtration, reabsorption, barrier, damage repair, and immunity. These cells are highly sensitive to DNA damage and inflammatory responses and may initiate pathological cascades upon injury (92).

In recent years, the role of cGAS-STING signaling pathway in kidney diseases has been gradually revealed. This pathway is activated by recognition of cytosolic DNA, initiating the innate immune response and inducing the production of inflammatory factors. In the kidney, abnormal activation of the cGAS-STING pathway is closely related to the pathogenesis of a variety of kidney diseases, especially in diseases such as AKI, CKD, and diabetic nephropathy (93–95). It is important to note that existing studies have demonstrated that activation of this pathway is highly specific to renal cells, with different cell types playing distinct roles in disease progression. For example, comprehensive RNA sequencing analyses of control and diseased kidneys from both human and mouse models reveal that TECs exhibit upregulated endogenous retroviral (ERV) expression under disease conditions. The resulting nucleic acid molecules can directly activate the intracellular STING pathway. Immunohistochemical analyses show that STING and its activated form, pSTING, are specifically enriched in TECs (96). This indicates that TECs are not passive “spectators” of inflammation but actively participate in and drive the early inflammatory response through the STING pathway. Therefore, the role of the cGAS-STING pathway in kidney diseases is cell type-dependent. Activation in TECs may represent the central mechanism for disease initiation and persistence, while involvement of immune cells accelerates the pathological process.

## Tubular epithelial cells

Renal tubular epithelial cells are rich in mitochondria, exhibit high metabolic activity, and rely predominantly on oxidative phosphorylation for energy production (97). TECs are the core of maintaining renal homeostasis through substance transport, endocrine regulation and remarkable repair capacity. However, TECs are also particularly vulnerable to external injuries such as ischemia-reperfusion, sepsis and renal toxicants. Their dysfunction directly leads to water and electrolyte disorders, toxin accumulation and systemic metabolic imbalance, which become the key initiating event of a variety of kidney diseases (98–100). Recent studies have revealed that severe or repeated injuries of proximal tubules may lead to permanent damage to their structure and loss of specific functions, which in turn trigger inflammatory infiltration, abnormal collagen deposition and subsequent pathological cascade reactions in renal interstitial (101). This process indicates that the damage of TECs is not only the main pathological driver of AKI progression, but also the core hub of CKD transformation. It is noteworthy that TECs can be activated under stress in the injured microenvironment, and actively mediate the infiltration and activation of neutrophils and macrophages by secreting chemokines, pro-inflammatory factors and signal regulatory molecules to amplify the inflammatory cascade (102–106). This process further accelerates the apoptosis of TECs, promotes the differentiation of fibroblasts into myofibroblasts, and eventually leads to the structural remodeling of the nephron and the collapse of the homeostasis of the renal microenvironment, leading to irreversible renal failure (107, 108).

### The cGAS-STING signaling pathway in TECs of AKI

In the above pathological process, TECs may cause mtDNA leakage or release of intranuclear DAMP-associated molecular patterns due to sustained damage, and activate the cGAS-STING signaling pathway (109). In the cisplatin-induced AKI, mitochondrial dysfunction in TECs leads to mtDNA leakage into the cytoplasm through the BAX pore, activates cGAS-STING signaling, and then drives proinflammatory factor transcription and neutrophil infiltration, while STING deficiency or knockdown attenuates inflammation (6).

In mice with renal ischemia-reperfusion injury (IRI), the expression level of STING is mainly increased in renal tubules, and STING deficiency can significantly reduce lipid peroxidation, tissue damage and renal function damage caused by IRI. Dynamin-related protein 1 (Drp1) -mediated mitochondrial fission triggers Bax translocation, which leads to mitochondrial membrane potential decrease and dsDNA release, and then activates the cGAS-STING pathway to trigger renal tubular inflammation. However, Drp1 inhibitor P110 inhibits this pathway and ameliorates renal tubular injury by blocking Drp1/Fis1 interaction (110). On the other hand, dual-specificity phosphatase 1 (DUSP1) deficiency in TECs promotes BAX mitochondrial translocation by enhancing JNK phosphorylation, causing mtDNA leakage and activating cGAS-STING, which aggravates AKI. This effect is reversed by JNK inhibitor or STING knockdown (111). Upregulation of STING in renal tubules after IRI can trigger ferritinophagy by binding to nuclear receptor coactivator 4 (NCOA4). It induces ferroptotic cell death, increases lipid ROS production and decreases GSH peroxidase 4 expression in TECs. The harmful effect of STING overexpression depends on the autophagic degradation of ferritin, leading to iron overload, lipid peroxidation, and driving ferroptosis. Conversely, knockdown or inhibition of STING markedly ameliorates ferroptosis and renal dysfunction (112). In addition, it has been found that the highly expressed small nucleolar RNA Snord3a in AKI regulates and activates the cGAS-STING signaling pathway by promoting transcription of the STING gene, thereby exacerbating iron overload-induced phenotypes, tubular cell death, and inflammatory responses (113).

STING also plays a central regulatory role in the occurrence and development of sepsis-associated AKI. In LPS-induced sepsis model, STING activation promotes mitochondrial reactive oxygen species excessive accumulation by inducing endoplasmic reticulum stress, thereby activating thioredoxin binding protein and NLRP3 binding. Finally, it drives the activation of NLRP3 inflammasome and causes pyroptosis. On the other hand, LPS-induced mtDNA released into the cytoplasm triggers the activation of NLRP3 inflammasome through the cGAS-STING axis, which promotes the release of inflammatory factors such as IL-1β and IL-18 and exacerbates kidney injury (114). These findings suggest that mtDNA leakage is the core link in the abnormal activation of cGAS-STING, and protecting mitochondrial integrity and regulating DNA leakage signals are key strategies for alleviating AKI.

### The cGAS-STING signaling pathway in TECs of CKD

Renal fibrosis is a common pathway for various CKD to develop into end-stage renal disease. Hyperglycolysis not only promotes energy metabolism imbalance, but also aggravates the excessive deposition of extracellular matrix and renal tubulointerstitial fibrosis by generating pro-fibrotic metabolites and activating TGF-β and other signals. During renal fibrosis, the loss of pyruvate carboxylase in the mitochondria of TECs or chronic hypoxia leads to the abnormal release of mtDNA into the cytoplasm, which activates the intracellular cGAS-STING pathway, and promotes the phosphorylation and nuclear translocation of the downstream transcription factor IRF3 (115). STING-IRF3 signal axis significantly enhances the level of glycolysis metabolism by up-regulating the expression of PFKFB3, a key glycolytic enzyme. Inhibition of STING or IRF3 can effectively reverse the abnormal glycolysis mediated by PFKFB3, thereby delaying the progression of renal fibrosis (116). Renal tubule-specific knockout of mitochondrial transcription factor A (TFAM) induces abnormal mtDNA packaging and release to the cytoplasm, continuously activates the cGAS-STING pathway, promotes cytokine secretion and immune cell recruitment, and eventually leads to progressive fibrosis and azotemia. In contrast, STING ablation inhibits fibrosis progression, confirming that TFAM limits pathological signals by isolating mtDNA (7).

In the development of CKD, endoplasmic reticulum stress promotes renal fibrosis through multiple signaling pathways, such as TGF-β, epithelial-mesenchymal transition and oxidative stress. Endoplasmic reticulum stress is a self-response mechanism of the body to various pathophysiological stimuli. Appropriate endoplasmic reticulum stress can restore endoplasmic reticulum homeostasis to maintain cell survival, while prolonged or severe endoplasmic reticulum stress may lead to programmed cell death, resulting in organ damage (117). The STING pathway enhances ER stress through a protein kinase R-like ER kinase (PERK)-mediated signaling cascade in TECs and subsequently increases fibrosis during renal injury. It has been found that the activation of STING in tubular cells after renal injury can directly physically interact with PERK, induce PERK phosphorylation and trigger ER stress, which in turn drives the expression of fibrosis-related genes. Clinical data further revealed spatial co-localization of STING-and PERK-dependent ER stress and fibrosis markers in renal fibrosis tissues. This implies that STING signaling plays an important role through ER stress during renal fibrosis (118, 119).

### The cGAS-STING signaling pathway in TECs of diabetic nephropathy

DKD is one of the main causes of CKD and end-stage renal disease. Its core pathological features are glomerular and tubulointerstitial damage caused by hyperglycemia and metabolic disorders, leading to inflammation, oxidative stress and mitochondrial damage, and the activation of iron death (120). STING protein in TECs has a dual pathogenetic mechanism in this process: On the one hand, high glucose or oxidative stress leads to mtDNA damage, activates the cGAS/STING signaling axis, drives the downstream TBK1/NF-κB pathway, promotes the release of pro-inflammatory factors such as IL-1β and TNF-α, and aggravates renal tubular inflammation and fibrosis (121). On the other hand, STING synergistically promotes ferroptosis and oxidative stress injury in TECs by increasing the ubiquitination and degradation of ferroportin FPN1, leading to intracellular iron accumulation and lipid peroxidation. Inhibition of STING can reduce inflammatory transmission and stabilize FPN1 to alleviate iron overload, thereby improving renal function injury (122).

**Figure 3:**
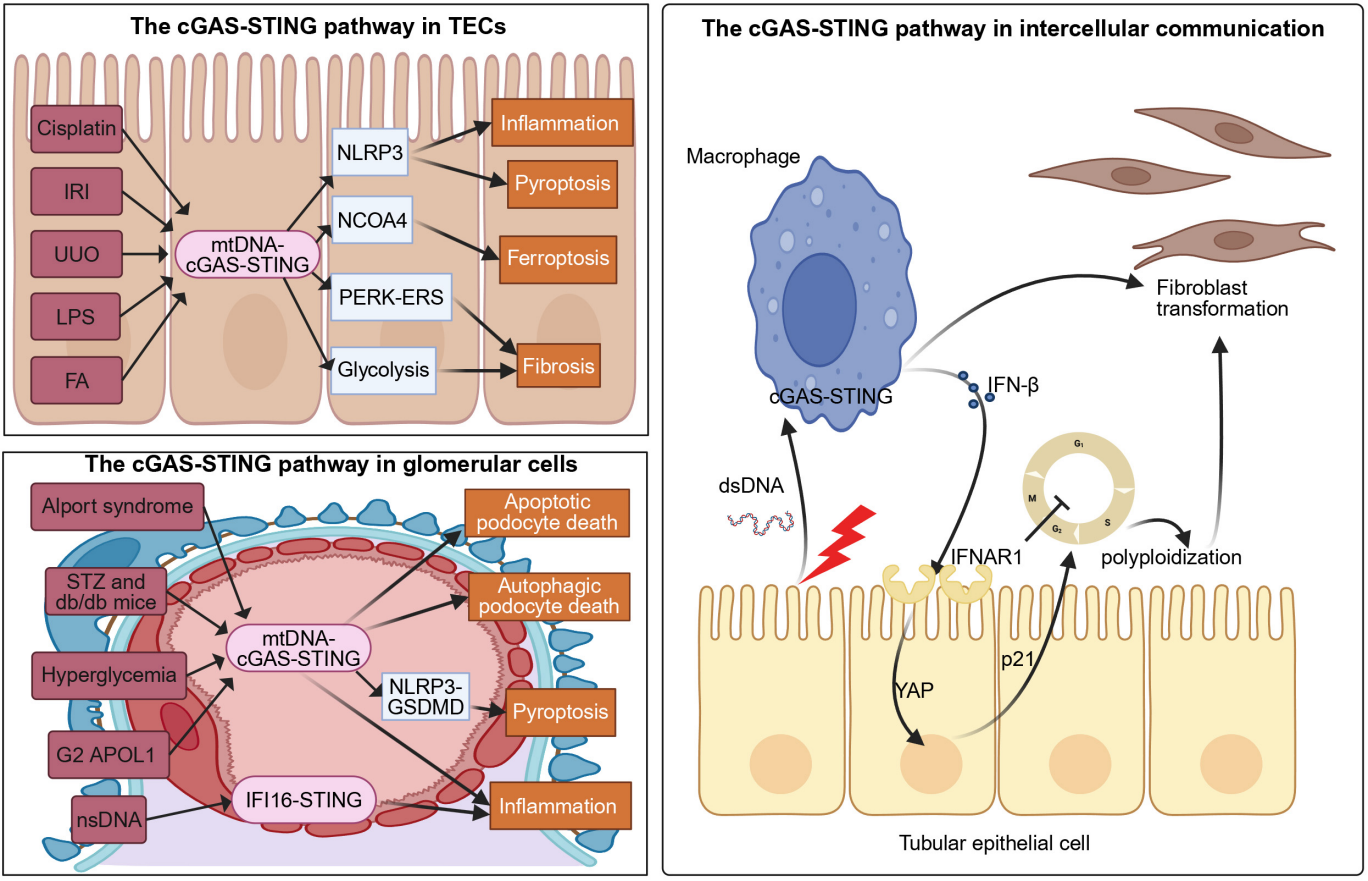
The specific role of the cGAS-STING signaling pathway in renal cells and its function in intercellular communication. The release of mtDNA is an important trigger for activating the cGAS-STING pathway. Upon activation, TECs predominantly undergo processes such as ferroptosis and fibrosis, whereas glomerular cells are more prone to pyroptosis and inflammatory responses (left). Injured TECs emit signals that activate macrophages, which subsequently secrete factors promoting fibroblast activation. Besides, these activated macrophages release IFN-β, which acts on TECs to induce polyploidization (right). NCOA4, nuclear receptor coactivator 4; LPS, lipopolysaccharide; PERK, protein kinase R-like ER kinase; HFD, high-fat diet; STZ, streptozotocin; APOL1, apolipoprotein L1; nsDNA, nucleosome-associated double-stranded DNA; IFI16, interferon-inducible protein 16; YAP, yes-associated protein. Figure created with BioRender.com.

Based on the studies discussed above, although activation of the STING pathway in TECs is consistently triggered by mitochondrial damage and mtDNA leakage, the downstream effects and ultimate cell fate exhibit significant heterogeneity. This variability is likely determined by the nature, intensity, and duration of the damage, as well as the metabolic microenvironment surrounding the TECs. For example, in acute injury models, STING activation rapidly links mitochondrial membrane permeabilization with the iron metabolism regulator NCOA4, leading to ferritin autophagy and lipid peroxidation, which strongly directs cell fate toward ferroptosis (112). In contrast, during chronic progression, sustained STING activation is more closely associated with endoplasmic reticulum stress and glycolytic dysregulation—pathways that tend to promote fibrosis (115, 116). During systemic inflammation, STING activation also establishes a positive feedback loop with the NLRP3 inflammasome, resulting in pyroptosis and the release of numerous inflammatory factors, thereby amplifying local damage into a systemic inflammatory storm (114, 123). A common theme is that inhibiting STING can alleviate kidney injury, indicating that it is a key target for therapeutic intervention in kidney diseases. However, the precise role of the STING signaling pathway in kidney diseases requires further investigation (see **Figure 3**).

## Glomerular cells

### Podocytes

Podocytes are specialized epithelial cells of the glomeruli that play a crucial role in maintaining the integrity of the glomerular filtration barrier. Similar to TEC, podocyte injury is also the core link of a variety of kidney diseases, such as minimal change disease and focal segmental glomerulosclerosis (FSGS). However, tubular injury is often triggered by ischemia and toxic substances, while podocytes are more susceptible to structural disintegration due to immune abnormalities, mechanical stress, or genetic defects (124).

### The cGAS-STING signaling pathway in podocytes of DKD

Recent studies have found that the cGAS-STING signaling pathway is involved in podocytes injury and proteinuria caused by diabetic nephropathy. On the one hand, after hyperglycemia or lipotoxicity induces mitochondrial damage in podocytes, BAX protein-mediated mtDNA leakage into the cytoplasm triggers TBK1 and NF-κB downstream of STING, leading to podocytes injury. Inhibition of STING or its downstream effector molecules in podocytes can significantly improve renal function damage caused by DKD. On the other hand, the up-regulation of STING expression in podocytes under high glucose environment can promote the activation of NLRP3. STING can also directly regulate NLRP3 inflammasome-dependent inflammatory response and pyroptosis, thereby causing pyroptosis and kidney injury (125). The specific activation of STING can lead to proteinuria and podocytes loss in C57BL/6 mice, while the baseline STING activity is abnormally increased in the glomerulus of db/db diabetic mice and Alport model mice. In DKD model, STING activation leads to proteinuria and glomerular filtration barrier disruption by promoting podocytes apoptosis. In Alport syndrome, however, STING mainly induces autophagy-dependent podocytes death (126).

### The cGAS-STING signaling pathway in podocytes of lupus nephritis and Alport syndrome

Apolipoprotein L1 (APOL1) alleles G1 and G2 are associated with faster progression to LN-associated end-stage renal disease in African Americans. APOL1 risk alleles (G1/G2) drive disease progression in podocytes nephropathy through multiple molecular mechanisms, in which STING pathway plays a central regulatory role. In the inflammatory microenvironment, nucleosome-associated double-stranded DNA fragments (nsDNA) directly induces APOL1 expression by activating the cGAS/IFI16-STING-IRF3 axis, and triggers IFN-β secretion to form an IFNAR-STAT1-IFI16 positive feedback loop, which significantly amplifies APOL1 expression (127). In animal models, the pathogenic effect of G2 APOL1 is closely related to the abnormal activation of STING and its downstream NLRP3-GSDMD pathway. Specific inhibition of STING or downstream inflammatory elements can effectively improve kidney injury (128). Notably, STING and hypoxia pathways functionally interact: in the DNA damage scenario, STING enhances APOL1 expression through an immune signaling cascade, while IFI16 acts independently of STING to assist HIF-1α in the hypoxic microenvironment (129). These research findings collectively illustrate the intricate regulatory network of APOL1 in podocytes. A combined therapeutic strategy targeting STING and its associated signaling pathways may provide a multidimensional approach for the treatment of APOL1-related nephropathy, but also confirm the central role of the STING pathway in integrating the innate immune response to mtDNA leakage and the NLRP3 inflammasome cascade in mediating disease pathology.

In summary, although the cGAS-STING pathway in podocytes is frequently activated by mitochondrial damage or abnormal DNA sensing, its downstream cellular outcomes primarily include apoptosis, pyroptosis, or autophagy-dependent cell death. Unlike TECs, which are mainly affected by metabolic insults such as ischemia and toxins, podocytes injury often results from immune complex deposition, complement activation, anti-DNA antibodies, or genetic defects. STING activation in podocytes establishes a robust positive feedback loop with the NLRP3-GSDMD axis, rendering pyroptosis a rapid and explosive form of cell death that directly compromises the filtration barrier and releases large amounts of inflammatory mediators (125). As terminally differentiated cells, podocytes rely heavily on autophagic flux to clear damaged organelles and maintain cytoskeletal integrity. Persistent STING activation may disrupt mTOR signaling, causing an imbalance or excessive activation of autophagy, which can lead to autophagy-dependent cell death (126). These differences stem from the unique biological characteristics of podocytes and the specific injurious environment they encounter within the glomerulus. Therefore, developing precise intervention strategies targeting the distinct death pathways in podocytes will be crucial for protecting the glomerular filtration barrier.

### Glomerular endothelial cells

Although a large number of studies have linked the activation of the cGAS-STING pathway with glomerular diseases, and the diseases are mainly concentrated in podocytes. However, several lines of evidence also suggest that other cells within the glomerulus are directly or indirectly affected by this pathway and may contribute to renal dysfunction. GECs are the first barrier of glomerular capillary wall, which can block blood cells and other visible components in blood and selectively trap macromolecular substances in blood. The luminal surface is covered with about 15nm thick polyanionic glycoprotein rich in sialic acid, which is also an important part of the barrier (130). When glomerular damage is severe, the endothelium is damaged, leading to neovascularization and sclerosis replacing the damaged area. GECs can also affect mesangial cells and epithelial cells after damage, and may affect the progression of kidney disease through their interaction (131).

### The cGAS-STING signaling pathway in GECs of kidney diseases

In the pathogenesis of DKD and FSGS, mitochondrial dysfunction in GECs is a key early event, which is manifested as mtDNA oxidative damage and ROS overproduction. In diabetic mice, mtDNA indirectly causes podocytes loss and proteinuria through GECs (132), and high glucose environment directly induces mitochondrial damage in GECs and activates STING signaling pathway (133). The FSGS transgenic model shows that TGFβR1 activation in podocytes can exacerbate GECs mitochondrial stress and mtDNA release, which leads to podocytes loss (134). In APOL1 nephropathy model, GECs mitochondrial autophagy enhancement is associated with mtDNA leakage and STING pathway activation (135). These studies have shown that GECs damage can also significantly affect the progression and repair of kidney diseases.

### Glomerular mesangial cells

GMCs are specialized adventitial cells that surround and restrict the vascular network within the glomerulus. These cells originate from the stromal mesenchyme and are distinct from nephron stem cells. They have various functions including synthesis and assembly of mesangial matrix, endocytosis and processing of plasma macromolecules, and control of glomerular hemodynamics by contraction of mesangial cells or release of vasoactive hormones (136).

### The cGAS-STING signaling pathway in GMCs of kidney diseases

The cGAS-STING pathway activation in mesangial cells research is still relatively limited, but there is evidence that mitochondrial damage in human mesangial cells cultured with galactose-deficient IgA from patients with IgA nephropathy may be related to downregulation of peroxisome proliferator-activated receptor α expression. High glucose environment promotes extracellular matrix (ECM) deposition, pro-inflammatory factor release and fibrosis process in mesangial cells by activating STING signal. The mechanism may involve mtDNA leakage caused by mitochondrial damage, and then trigger cGAS-STING pathway (137).

These findings suggest a multicellular cooperative mechanism by which the cGAS-STING pathway mediates glomerular injury. Inhibition of STING can target a variety of pathological processes, but its cell type specificity and selectivity of downstream effector molecules still need to be further studied.

## Renal interstitial fibroblasts and macrophages

Abnormal glomerular and interstitial regions and activation of myofibroblasts that promote excessive ECM protein deposition are typical markers of renal fibrosis and exacerbate the severity of renal injury. Recent findings have identified distinct populations of myofibroblasts as the primary source of ECM in scar tissue formation (138). However, the origin of fibroblasts in renal fibrosis remains the subject of debate. Advances in lineage tracing and immunofluorescence microscopy have revealed that myofibroblasts may be derived from multiple sources, such as activated renal fibroblasts, pericytes, epithelial-to-mesenchymal transition, endothelial-to-mesenchymal transition, bone marrow-derived cells, and fibroblasts. The residual nephron in CKD is in a hypermetabolic state, with increased oxygen consumption and oxygen free radicals. At the same time, proteinuria, inflammatory cell infiltration, activation of renal interstitial fibroblasts, and excessive accumulation of extracellular matrix in the renal interstitium can cause renal tubular cell damage (139). During the progression of nephropathy, fibroblasts and renal immune cells work together to drive tissue damage and fibrosis through complex interactions. At present, there are considerable achievements in studying the activation of cGAS-STING signaling pathway in renal macrophages to aggravate the progression of nephropathy.

### The cGAS-STING signaling pathway in fibroblasts and macrophages of kidney diseases

In a model of obstructive nephrosis, double-stranded DNA released from damaged TECs activates the cGAS-STING pathway in macrophages, triggering inflammatory signaling cascades, thereby inducing fibroblast activation and fibrotic phenotype transformation (140). In the folate-induced renal injury model, blocking STING/TBK1 signaling axis can significantly inhibit the activation of bone marrow-derived fibroblasts and the transdifferentiation of macrophages into myofibroblasts, thereby reducing collagen deposition and the formation of renal fibrosis lesions (141). By mediating the phenotypic transformation of macrophages and the effect function of fibroblasts, STING pathway has become the core regulatory hub connecting the innate immune response and the progression of renal fibrosis. In addition, macrophages recognize intracellular damage-associated molecular patterns after kidney injury, activate the cGAS-STING-TBK1-IRF3 signaling axis, and stimulate the synthesis and release of IFN-β. The released IFN-β binds to the IFNAR1 receptor on TECs, triggering the interaction between inorganic pyrophosphatase and Yes-associated protein (YAP). This interaction results in the dephosphorylation of YAP, which subsequently translocates into the nucleus, upregulating the expression of p21, arresting the G2/M transition, and inducing polyploidy. Notably, delayed inhibition of STING pathway (intervention on day 4 after AKI) effectively interrupted the pro-fibrotic signaling between macrophages and epithelial cells and alleviated persistent renal fibrosis (12). This mechanism directly links innate immune response to tissue abnormal repair, suggesting that targeting macrophage STING pathway may become a new strategy to delay AKI-CKD transition.

In summary, the role of the cGAS-STING pathway in the kidney constitutes a complex multicellular network. Energy-demanding and metabolically active cells, such as TECs and podocytes, are more susceptible to death due to metabolic disruption or imbalances in organelle homeostasis caused by STING activation. GECs and GMCs are primarily involved in upregulating the ECM synthetic gene program. Activation of STING in macrophages transforms them from immune sentinels into potent producers of inflammatory factors, amplifying classic IFN-I and inflammasome responses. Moreover, macrophages regulate the cell cycle and phenotype of TECs both directly and indirectly through paracrine signaling, thereby promoting the progression from AKI to CKD. Fibroblasts and myofibroblasts serve as the ultimate effectors of fibrosis downstream of the STING pathway. Although mitochondrial damage and mtDNA leakage are common upstream triggers across cell types, the pathological roles of different cells following STING activation vary due to their distinct intrinsic functions, characteristics, and interactions with the microenvironment.

## Therapeutic potential of the cGAS-STING pathway in kidney disease

In recent years, as the core inflammatory signal axis of the innate immune system, the cGAS-STING pathway has emerged as a promising therapeutic target for the treatment of kidney diseases due to its key role in regulating the release of IFN-I and proinflammatory factors. Inhibition of the cGAS-STING pathway by specific chemical molecules is expected to improve kidney diseases (142, 143). Upstream, cGAS functions as a DNA sensor that initiates downstream signaling pathways through the synthesis of cGAMP. Downstream, the recruitment and phosphorylation of TBK1 and IRF3 are critical events driving immune activation. Also, the mechanisms underlying the conformational changes and post-translational modifications of STING following ligand recognition have been thoroughly elucidated. The strategies to inhibit STING by inhibiting palmitoylation and occupying CDN pockets have been confirmed (144).

### Small molecule inhibitors

Acetylation modification is a key molecular event controlling cGAS activity, and acetylation at K384, K394 and K414 has been found to inhibit cGAS activation (145). Vincent et al. discovered a series of active compounds through high-throughput screening. The representative compound RU.521 showed potent cellular activity and selectivity (146). Pharmacological inhibition of cGAS by RU.521 reduced macrophage proinflammatory activation, inhibited myofibroblast formation, and attenuated renal fibrosis after obstructive injury (140).

SN-011, as a specific STING inhibitor binding to CDN-binding pockets, is able to compete with cGAMP binding STING (147). SN-011 could regulate the NF-κB and MAPK pathways, inhibit the expression of inflammatory factors, and reduce the ROS release induced by cisplatin in the cell model. In addition, SN-011 blocked the nuclear translocation of NF-κB p65, further reduced inflammatory response, improved mouse survival and alleviated renal dysfunction (14). STING palmitoylation is one of the key processes that promote STING aggregation at the Golgi apparatus and subsequent recruitment of STING downstream signaling molecules. As a core regulatory protein, STING not only receives damaged signals from host DNA, but also responds to gene mutations and endoplasmic reticulum stress, thus participating in the regulation of a variety of autoimmune and inflammatory diseases. From this point of view, targeting STING may be preferable to targeting upstream cGAS or downstream TBK1 in drug discovery. C-176 and H-151 covalently bind to Cys88 or Cys91 residues in the transmembrane region of STING protein, block its palmitoylation modification, and inhibit the transport and oligomerization of STING from endoplasmic reticulum to Golgi (143). H-151 attenuates renal inflammation and extracellular matrix deposition by blocking STING palmitoylation in TECs or interfering with its downstream TBK1/IRF3 signaling pathway (15, 148, 149). In addition, C-176 intervention can inhibit the polarization of M0 macrophages to M1 macrophages, promote their polarization to M2 macrophages, and reduce the expression of pro-inflammatory cytokines such as IL-6 and TNF-α at the protein and gene levels, thereby improving the sepsis induced AKI (150). The above studies indicate that small molecule inhibitors of the cGAS-STING pathway can improve the abnormal activation of the cGAS-STING pathway caused by mtDNA leakage, alleviate TEC damage, macrophage infiltration and fibrosis in a variety of kidney disease models. It is expected that highly effective and low-toxicity cGAS-STING pathway inhibitors will be developed and eventually applied to clinical practice in the near future.

### Natural compounds

Although significant progress has been made in the treatment of synthetic small molecule inhibitors targeting the cGAS-STING pathway, their long-term safety, insufficient targeting, and potential risk of immunosuppression still need to be optimized. At the same time, the multitarget mechanisms of traditional Chinese medicine compounds may provide a natural molecular library for the development of STING inhibitors (151). The introduction of nanotechnology is expected to break through the limitations of existing drugs through precision delivery and intelligent drug delivery systems, and become an important direction for the next generation of kidney disease treatment strategies (152). Polydatin (PD) is a natural compound that directly binds to the STING protein, promoting its degradation through the proteasome pathway. This action inhibits the production of pro-inflammatory and fibrotic factors in GMCs under high-glucose conditions. In diabetic mice, PD also suppresses the STING pathway and mitigates pathological changes associated with renal inflammatory fibrosis (153). Total glucoside of Paeoniae alba (TGP) is the main component of Paeoniae alba, which has anti-inflammatory, immune regulation, liver protection and other functions. It is often used in the treatment of chronic hepatitis, rheumatoid arthritis and senile diseases (154). TGP significantly inhibited the activation of the cGAS-STING signaling pathway induced by various cGAS-STING agonists in mouse BMDMs and THP-1 cells by blocking the interaction between STING and IRF3, thereby reducing the production of IFN-β and inflammatory factors (155). Urolithin A (UroA), as the main metabolite of ellagitannin, has intrinsic biological effects similar to or higher than that of the parent compound, such as anti-inflammatory activity and promoting autophagy (156). In fructose-induced hyperuricemic nephropathy in mice, UroA inhibits the STING-NLRP3 inflammatory axis by activating Parkin-dependent mitophagy, thereby reducing oxidative stress and renal tubular injury. Furthermore, in HK-2 cells, silencing the Parkin gene impairs the inhibitory effect of UroA on STING-NLRP3 activation (157). Astragalus polysaccharide (APS) is a polysaccharide extracted from Astragalus. It has anti-inflammatory, antioxidant, immunomodulatory, anti-aging and anti-tumor functions (158). In a mouse model of AKI induced by rhabdomyolysis, APS inhibits the activation of the cGAS-STING pathway in macrophages, reduces the polarization of macrophages toward the M1 phenotype, mitigates kidney damage caused by the inflammatory microenvironment, and preserves renal function. Additionally, when co-cultured with M1-type macrophages, APS demonstrates an anti-apoptotic effect on MPC5 cells (159). Natural compounds, such as TGP, UroA and APS, have shown significant therapeutic effects on kidney diseases by directly inhibiting STING signaling or regulating its upstream activators. The synergistic effect of these components across pathways provides new ideas to solve the limitations of single target inhibition.

### Traditional Chinese medicine formulae

In addition to the targeting of a single component, TCM formulae, through a variety of active ingredients and synergies, can realize the dynamic regulation of STING pathway (160–162). For example, Shenqi Fuzheng Injection (SQFZ), a traditional Chinese medicine injection composed of extracts of Codonopsis and Astragalus, improves cisplatin induced AKI. SQFZ reduces cisplatin-induced apoptosis and mtDNA damage, and reverses cisplatin-induced cGAS-STING signaling pathway activation (160). Astragalus Danshen Decoction (HDD), consisting of Astragalus and Salvia miltiorrhiza, dose-dependently inhibits STING pathway activation, thereby improving renal fibrosis in adenine-induced CKD mouse models (161). Zhen Wu Decoction (ZWD) is a prescription from the classical text “Treatise on Exogenous Febrile Disease”. Renal fibrosis can be limited by maintaining mitochondrial integrity, improving oxidative phosphorylation, and restoring tubular bioenergy capacity (163). These traditional Chinese medicine compounds inhibit the excessive activation of STING pathway through a variety of ways, thereby restoring renal cell homeostasis, and may be better adapted to the complex pathological network of the disease.

### Nano-delivery systems

Based on the unique drug carrying ability, precise delivery characteristics and surface functional modifiability of nanomaterials, the construction of an intelligent nanomedicine delivery system targeting STING pathway has made good progress in kidney disease research. A variety of natural or synthetic nano-carriers are loaded with drugs with antioxidant activity to construct intelligent delivery systems, which systematically solve the problems of low drug solubility and poor targeting. These nanodrugs can enhance the renal accumulation effect at the lesion site, precisely remove ROS, protect mitochondrial functional integrity, reduce DNA damage and mtDNA leakage, and then block the abnormal activation of cGAS-STING pathway. In a variety of AKI models, such as cisplatin and excess folic acid, nano-therapy systems effectively reverse abnormal renal function indicators and repair pathological damage to kidney tissue by regulating the multi-stage pathological axis of oxidative stress, mitochondrial damage, DNA leakage and STING activation (164–170). Nanocrystals can not only realize the spatiotemporal controlled release of drugs in kidney lesions, but also break through the biological barrier through active targeting strategy and dually regulate the excessive activation of cGAS-STING signaling pathway, thereby inhibiting the inflammatory pyroptosis and fibrosis process of TECs. It provides an innovative strategy for the development of precise treatment of kidney disease by targeting immune microenvironment regulation.

Overall, small molecule inhibitors targeting the cGAS-STING pathway exhibit direct actions with high specificity. Nevertheless, their clinical application may be constrained by potential off-target effects, widespread toxicity, and the absence of fundamental targeting capabilities toward damaged renal cells (171). In contrast, natural compounds and TCM formulations primarily exert therapeutic effects through indirect, multi-target modulation. Their well-documented antioxidant, anti-inflammatory, and mitochondrial protective properties likely suppress pathway activation by mitigating upstream stimuli rather than through direct, high-affinity interactions with core pathway components. Although such broad-range activity may offer advantages in addressing complex disease networks, it simultaneously complicates elucidation of precise mechanisms and limits therapeutic specificity. Nano-delivery systems present a promising technological strategy to surmount the limitations associated with both synthetic and natural agents (172). By enabling spatiotemporally controlled release and enhanced accumulation at sites of renal injury, nanotechnology holds the potential to enhance the effectiveness and safety profiles of both direct inhibitors and indirect modulators. Future advancements in developing ligands with cell-specific targeting capabilities, combined with potent direct STING inhibitors encapsulated within intelligent nanocarriers, may finally assist in realizing truly precise and effective therapeutic interventions (see **Table 2**).

**Table 2:** Therapeutic potential of the cGAS-STING pathway in kidney disease.

Strategy category	Representative drugs	Effect (direct or indirect*)	Molecular mechanism	Disease	Model/ treatment	Duration	Cell lines	Key effects	Refs
Small- Molecule Inhibitors	RU.521	Direct	Inhibits cGAS activation via acetyltransferase modulation	CKD	Animal: UUO; Cell: H_2_O_2_	10 days	Mouse bone marrow-derived macrophage and TEC	Macrophage proinflammatory activation↓; Myofibroblast formation↓; Fibrosis↓	(140)
	SN-011	Direct	Competes with cGAMP for STING's CDN-binding pocket	AKI	Animal: Cisplatin; Cell: Cisplatin	64 h	HK-2	Inflammatory↓; ROS release↓	(14)
	H-151	Direct	Covalently binds the STING Cys88/91 residues, blocking palmitoylation and Golgi transport	AKI	Animal: IRI,; Cell: extracellular cold-inducible RNA-binding protein	1 days	Primary TEC	Inflammation and apoptosis↓; Mitochondrial injury↓	(15, 148, 149)
					Animal: Cisplatin; Cell: -	3 days	–		
					Animal: LPS;	12 h	–		
	C-176	Direct	Covalently binds the STING Cys88/91 residues, blocking palmitoylation and Golgi transport	AKI	Animal: LPS; Cell: LPS and IFN-γ	3 days	Mouse bone marrow-derived macrophage	M0→M1 polarization↓; M0→M2 polarization↑; Inflammation↓	(150)
Natural Compounds	Polydatin (PD)	Direct	Combines with STING and promotes its protein degradation	DKD	Animal: STZ-HFD; Cell: high glucose	10 weeks	Primary rat glomerular mesangial cell	ECM↓; Inflammatory↓; Fibrosis↓	(153)
	Total glucoside of Paeoniaealba (TGP)	Indirect	Disrupts STING-IRF3 interaction	AKI	Animal: LPS; Cell: interferon stimulatory DNA, cGAMP	7 days	Mouse bone marrow-derived macrophage and THP-1	Inflammatory↓	(155)
	Urolithin A (UroA)	Indirect	Restores PINK1/Parkin- mediated mitophagy and Inhibits STING-NLRP3 axis	CKD	Animal: Hyperuricemia nephropathy induced by fructose feeding; Cell: Uric Acid	8 weeks	HK-2	Inflammation↓; mitophagy↑	(157)
	Astragalus polysaccharide (APS)	Indirect	Inhibition of the activation of cGAS-STING pathway in macrophages	AKI	Animal: 50% glycerol-induced rhabdomyolysis; Cell: LPS and IFN-γ	1 day	Raw264.7 and MPC-5	M0→M1 polarization↓; Inflammation and apoptosis↓	(159)
TCM Formulae	Shenqi Fuzheng Injection (SQFZ)	Indirect	Reduces mtDNA damage and oxidative stress	AKI	Animal: Cisplatin; Cell: Cisplatin	18 days	4T1 and HK-2	Inflammation and apoptosis↓ ;mtDNA damage↓	(160)
	Huangqi-Danshen decoction (HDD)	Indirect	Regulation of cGAS-STING signaling through targeting SCD1	CKD	Animal: 0.2% adenine feed; Cell: TGF-β1	28 days	Primary TEC	Renal fibrosis↓	(161)
	Compound Danshen Dripping Pill (CDDP)	Direct	Inhibits the phosphorylation of IRF3 and eliminates the STING-TBK1 interaction	CKD	Animal: High-fat feeding; Cell: interferon stimulatory DNA, cGAMP	12 weeks	Mouse bone marrow-derived macrophage and THP-1	Inflammatory↓	(162)
	.Zhen Wu Decoction (ZWD)	Indirect	Protects mitochondrial DNA integrity	CKD	Animal: UUO and Folic Acid; Cell: TGF-β1	7 days	HK-2	Oxidative stress and inflammation↓; mtDNA leakage↓; Renal fibrosis↓	(163)
Nano-Delivery Systems	Baicalein-loaded silk fibroin peptide nanofibers (SFP/BA NFs)	Indirect	Increases the uptake and mitochondrial localization of drugs and inhibits DNA damage	AKI	Animal: Cisplatin; Cell: Cisplatin	3 days	HK-2	ROS and mitochondrial membrane potential disruption↓; Abnormal changes of antioxidant enzymes↓; Mitochondrial DNA damage↓	(164)
	Kolliphor HS15-based myricetin-loaded (HS15-Myr) nanomicelles	Indirect	Inhibits the accumulation of reactive oxygen species, reduction of mitochondrial membrane potential, and DNA damage	AKI	Animal: Cisplatin; Cell: Cisplatin	2 days	HK-2	The activities of antioxidant enzymes↑;Oxidative stress and inflammation↓	(165)
	Fucoidan-ferulic acid nanoparticles(FA/FUNPs)	Indirect	Inhibits DNA damage	AKI	Animal: Cisplatin; Cell: Cisplatin	1 day	HK-2	Inflammation↓; MDA activity↓; GSH and SOD activity↑	(166)
	Silk fibroin peptide self-assembled nanofibers delivered naringenin (SFP/NGN NFs)	Indirect	Activates mitophagy and inhibites mtDNA release	AKI	Animal: Cisplatin; Cell: Cisplatin	3 days	HK-2	Mitochondrial damage, mitophagy and mtDNA release↓; Inflammation↓	(167)
	Naringenin loaded fucoidan/polyvinylpyrrolidone nanoparticles (FU/PVP-NAR)	Indirect	Inhibits DNA damage	AKI	Animal: Folic Acid; Cell: Folic Acid	4 days	HK-2	ROS accumulation and MMP disruption↓	(168)
	Fucoidan-proanthocyanidins nanoparticles (FU/PCNPs)	Indirect	Reduces mitochondrial damage, activates mitophagy, and inhibits mtDNA release	AKI	Animal: Cisplatin; Cell: Cisplatin	3 days	HK-2	Mitochondrial damage, mitophagy and mtDNA release↓; Inflammation↓	(169)
	Hierarchical-targeting antioxidant nanodrug (HAND)	Indirect	Targets injured PTECs to protect mitochondria and nuclei	AKI	Animal: 50% glycerol-induced rhabdomyolysis; Cell: H_2_O_2_	1 day	HK-2	Mitochondrial function↑; Apoptosis↓ DNA oxidation and breakage↓	(170)

*Direct: By directly acting on the cGAS/STING protein. Indirect: By reducing the upstream triggering factors.↑ indicates an increase or elevation in the expression level compared to the control group or baseline; ↓ indicates a decrease or reduction.

## Conclusions and future perspectives

The cGAS-STING signaling pathway is a central component of the innate immune system. It plays a crucial role in host defense by recognizing microbial DNA and triggering interferon responses to combat pathogen invasion. However, in non-infectious kidney diseases, abnormal self-DNA released from mitochondrial or tissue damage activates this pathway, leading to sustained injury in various renal cells and thereby contributing to the progression of multiple kidney disorders. In TECs, this pathway induces endoplasmic reticulum stress, glycolytic reprogramming and ferroptosis through mtDNA leakage, driving the transformation of AKI to CKD. In podocytes, abnormal activation of STING mediates pyroptosis and autophagy-dependent death through NLRP3 inflammasome, leading to proteinuria and filtration barrier destruction. In renal interstitial macrophages, STING stimulates the secretion of IFN-β, preventing the transformation of TECs from the G2/M phase and inducing polyploidy formation. Currently, small molecule inhibitors targeting this pathway, such as H-151 and SN-011, inhibit the inflammatory cascade by blocking STING palmitoylation or competitively binding to the cGAMP pocket. Natural compounds and traditional Chinese medicine compounds synergistically regulate oxidative stress and mitochondrial function through multiple targets. Nanodrug delivery systems, such as alginate-resveratrol nanoparticles, have shown significant efficacy in a variety of nephropathy models by precisely targeting the renal lesion site to regulate DNA leakage and STING activation.

Although progress has been made in the mechanism research and targeted therapy of cGAS-STING pathway in nephropathy, the existing studies mostly focus on renal tubules and podocytes, while there is insufficient research on the mechanism of STING in GECs and GMCs. If single-cell RNA-seq data from human and mouse kidneys—both healthy and affected by various kidney diseases—can be collected and the expression patterns of STING and its pathway genes systematically analyzed, it will help clarify the relative expression levels and activation states of the cGAS-STING pathway across different renal cell clusters. Therefore, future research on the cGAS-STING pathway should move beyond the traditional Cre-lox classification model and shift toward cell function localization and mechanism-driven cell fate analysis based on unbiased single-cell data. Additionally, since activation of the cGAS-STING pathway leads to distinct fate outcomes in different renal cells, the underlying molecular determinants remain unclear. Future studies could compare transcriptomic, proteomic, and metabolomic changes in various renal cell lines following STING activation, identify differential pathways, and focus on molecules that may govern cell fate. Finally, although small molecule inhibitors targeting the cGAS-STING pathway are highly effective, they lack precise targeting of damaged cells. The development of nanodelivery systems could address challenges such as low drug solubility and poor targeting at injury sites. This includes designing delivery systems that target specific cell subpopulations and developing small molecule drugs that regulate specific functional branches of STING rather than STING itself. Meanwhile, exploring biomarkers that reflect the activation status of STING in specific cells is crucial for achieving patient stratification and personalized treatment. Future research needs to bridge the gap between specific mechanisms and clinical translation, combining precision medical technology and innovation, promoting cGAS-STING targeted therapy from basic to clinical and providing innovative treatment for kidney disease.

## References

[1] Barber GN . Sting: infection, inflammation and cancer. Nat Rev Immunol. (2015) 15:760–70. doi: 10.1038/nri3921 26603901 PMC5004891

[2] Wu J Sun L Chen X Du F Shi H Chen C . Cyclic gmp-amp is an endogenous second messenger in innate immune signaling by cytosolic DNA. Sci (New York NY). (2013) 339:826–30. doi: 10.1126/science.1229963 23258412 PMC3855410

[3] Tanaka Y Chen ZJ . Sting specifies irf3 phosphorylation by tbk1 in the cytosolic DNA signaling pathway. Sci Signaling. (2012) 5:ra20. doi: 10.1126/scisignal.2002521 22394562 PMC3549669

[4] Gulen MF Koch U Haag SM Schuler F Apetoh L Villunger A . Signalling strength determines proapoptotic functions of sting. Nat Commun. (2017) 8:427. doi: 10.1038/s41467-017-00573-w 28874664 PMC5585373

[5] Zhao Q Wei Y Pandol SJ Li L Habtezion A . Sting signaling promotes inflammation in experimental acute pancreatitis. Gastroenterology. (2018) 154:1822–1835.e2. doi: 10.1053/j.gastro.2018.01.065 29425920 PMC6112120

[6] Maekawa H Inoue T Ouchi H Jao TM Inoue R Nishi H . Mitochondrial damage causes inflammation via cgas-sting signaling in acute kidney injury. Cell Rep. (2019) 29:1261–1273.e6. doi: 10.1016/j.celrep.2019.09.050 31665638

[7] Chung KW Dhillon P Huang S Sheng X Shrestha R Qiu C . Mitochondrial damage and activation of the sting pathway lead to renal inflammation and fibrosis. Cell Metab. (2019) 30:784–799.e5. doi: 10.1016/j.cmet.2019.08.003 31474566 PMC7054893

[8] Hill NR Fatoba ST Oke JL Hirst JA O'Callaghan CA Lasserson DS . Global prevalence of chronic kidney disease - a systematic review and meta-analysis. PloS One. (2016) 11:e0158765. doi: 10.1371/journal.pone.0158765 27383068 PMC4934905

[9] Li J Hu Z . Research progress on damage-associated molecular patterns in acute kidney injury. Front Immunol. (2025) 16:1590822. doi: 10.3389/fimmu.2025.1590822 40709185 PMC12288145

[10] Ma L Liu D Yu Y Li Z Wang Q . Immune-mediated renal injury in diabetic kidney disease: from mechanisms to therapy. Front Immunol. (2025) 16:1587806. doi: 10.3389/fimmu.2025.1587806 40534883 PMC12173918

[11] Gao L Zhang J Yang T Jiang L Liu X Wang S . Sting/acsl4 axis-dependent ferroptosis and inflammation promote hypertension-associated chronic kidney disease. Mol Therapy: J Am Soc Gene Ther. (2023) 31:3084–103. doi: 10.1016/j.ymthe.2023.07.026 37533255 PMC10556226

[12] Wang Y Lan Q Li F Xiong J Xie H Gong S . Macrophage-derived type 1 ifn, renal tubular epithelial cell polyploidization, and aki-to-ckd transition. J Am Soc Nephrology: JASN. (2025) 36:766–80. doi: 10.1681/asn.0000000577 39665291 PMC12059107

[13] Bi X Du C Wang X Wang XY Han W Wang Y . Mitochondrial damage-induced innate immune activation in vascular smooth muscle cells promotes chronic kidney disease-associated plaque vulnerability. Advanced Sci (Weinheim Baden-Wurttemberg Germany). (2021) 8:2002738. doi: 10.1002/advs.202002738 33717842 PMC7927614

[14] Li Z Mao C Zhao Y Zhao Y Yi H Liu J . The sting antagonist sn-011 ameliorates cisplatin induced acute kidney injury via suppression of sting/nf-κb-mediated inflammation. Int Immunopharmacol. (2025) 146:113876. doi: 10.1016/j.intimp.2024.113876 39709905

[15] Hu Z Zhang F Brenner M Jacob A Wang P . The protective effect of h151, a novel sting inhibitor, in renal ischemia-reperfusion-induced acute kidney injury. Am J Physiol Renal Physiol. (2023) 324:F558–67. doi: 10.1152/ajprenal.00004.2023 37102684 PMC10228668

[16] Civril F Deimling T de Oliveira Mann CC Ablasser A Moldt M Witte G . Structural mechanism of cytosolic DNA sensing by cgas. Nature. (2013) 498:332–7. doi: 10.1038/nature12305 23722159 PMC3768140

[17] Sun L Wu J Du F Chen X Chen ZJ . Cyclic gmp-amp synthase is a cytosolic DNA sensor that activates the type I interferon pathway. Sci (New York NY). (2013) 339:786–91. doi: 10.1126/science.1232458 23258413 PMC3863629

[18] Barber GN . Cytoplasmic DNA innate immune pathways. Immunol Rev. (2011) 243:99–108. doi: 10.1111/j.1600-065X.2011.01051.x 21884170

[19] Zhao B Xu P Rowlett CM Jing T Shinde O Lei Y . The molecular basis of tight nuclear tethering and inactivation of cgas. Nature. (2020) 587:673–7. doi: 10.1038/s41586-020-2749-z 32911481 PMC7704945

[20] Pathare GR Decout A Glück S Cavadini S Makasheva K Hovius R . Structural mechanism of cgas inhibition by the nucleosome. Nature. (2020) 587:668–72. doi: 10.1038/s41586-020-2750-6 32911482

[21] Kujirai T Zierhut C Takizawa Y Kim R Negishi L Uruma N . Structural basis for the inhibition of cgas by nucleosomes. Sci (New York NY). (2020) 370:455–8. doi: 10.1126/science.abd0237 32912999 PMC7584773

[22] Boyer JA Spangler CJ Strauss JD Cesmat AP Liu P McGinty RK . Structural basis of nucleosome-dependent cgas inhibition. Sci (New York NY). (2020) 370:450–4. doi: 10.1126/science.abd0609 32913000 PMC8189757

[23] Michalski S de Oliveira Mann CC Stafford CA Witte G Bartho J Lammens K . Structural basis for sequestration and autoinhibition of cgas by chromatin. Nature. (2020) 587:678–82. doi: 10.1038/s41586-020-2748-0 32911480

[24] Cao D Han X Fan X Xu RM Zhang X . Structural basis for nucleosome-mediated inhibition of cgas activity. Cell Res. (2020) 30:1088–97. doi: 10.1038/s41422-020-00422-4 33051594 PMC7784699

[25] Wu S Gabelli SB Sohn J . The structural basis for 2'-5'/3'-5'-cgamp synthesis by cgas. Nat Commun. (2024) 15:4012. doi: 10.1038/s41467-024-48365-3 38740774 PMC11091121

[26] Liu S Yang B Hou Y Cui K Yang X Li X . The mechanism of sting autoinhibition and activation. Mol Cell. (2023) 83:1502–1518.e10. doi: 10.1016/j.molcel.2023.03.029 37086726

[27] Zhang C Shang G Gui X Zhang X Bai XC Chen ZJ . Structural basis of sting binding with and phosphorylation by tbk1. Nature. (2019) 567:394–8. doi: 10.1038/s41586-019-1000-2 30842653 PMC6862768

[28] Bowie A . The sting in the tail for cytosolic DNA-dependent activation of irf3. Sci Signaling. (2012) 5:pe9. doi: 10.1126/scisignal.2002919 22394560

[29] Yum S Li M Fang Y Chen ZJ . Tbk1 recruitment to sting activates both irf3 and nf-κb that mediate immune defense against tumors and viral infections. PNAS. (2021) 118(14):e2100225118. doi: 10.1073/pnas.2100225118 33785602 PMC8040795

[30] Yamamoto M Gohda J Akiyama T Inoue JI . Tnf receptor-associated factor 6 (traf6) plays crucial roles in multiple biological systems through polyubiquitination-mediated nf-κb activation. Proc Japan Acad Ser B Phys Biol Sci. (2021) 97:145–60. doi: 10.2183/pjab.97.009 33840674 PMC8062261

[31] Jiang S Li H Zhang L Mu W Zhang Y Chen T . Generic Diagramming Platform (GDP): a comprehensive database of high-quality biomedical graphics. Nucleic Acids Res. (2025) 53(D1):D1670–6. doi: 10.1093/nar/gkae973 PMC11701665 39470721

[32] Liu N Pang X Zhang H Ji P . The cgas-sting pathway in bacterial infection and bacterial immunity. Front Immunol. (2021) 12:814709. doi: 10.3389/fimmu.2021.814709 35095914 PMC8793285

[33] Shi C Yang X Liu Y Li H Chu H Li G . Zdhhc18 negatively regulates cgas-mediated innate immunity through palmitoylation. EMBO J. (2022) 41:e109272. doi: 10.15252/embj.2021109272 35438208 PMC9156970

[34] Shen X Sun C Cheng Y Ma D Sun Y Lin Y . Cgas mediates inflammation by polarizing macrophages to m1 phenotype via the mtorc1 pathway. J Immunol (Baltimore Md 1950). (2023) 210:1098–107. doi: 10.4049/jimmunol.2200351 36881861

[35] Watson RO Bell SL MacDuff DA Kimmey JM Diner EJ Olivas J . The cytosolic sensor cgas detects mycobacterium tuberculosis DNA to induce type I interferons and activate autophagy. Cell Host Microbe. (2015) 17:811–9. doi: 10.1016/j.chom.2015.05.004 26048136 PMC4466081

[36] Hansen K Prabakaran T Laustsen A Jørgensen SE Rahbæk SH Jensen SB . Listeria monocytogenes induces ifnβ expression through an ifi16-, cgas- and sting-dependent pathway. EMBO J. (2014) 33:1654–66. doi: 10.15252/embj.201488029 24970844 PMC4194099

[37] Seebach E Sonnenmoser G Kubatzky KF . Staphylococcus aureus planktonic but not biofilm environment induces an ifn-β macrophage immune response via the sting/irf3 pathway. Virulence. (2023) 14:2254599. doi: 10.1080/21505594.2023.2254599 37655977 PMC10496530

[38] Fischer K Tschismarov R Pilz A Straubinger S Carotta S McDowell A . Cutibacterium acnes infection induces type I interferon synthesis through the cgas-sting pathway. Front Immunol. (2020) 11:571334. doi: 10.3389/fimmu.2020.571334 33178195 PMC7593769

[39] Wassermann R Gulen MF Sala C Perin SG Lou Y Rybniker J . Mycobacterium tuberculosis differentially activates cgas- and inflammasome-dependent intracellular immune responses through esx-1. Cell Host Microbe. (2015) 17:799–810. doi: 10.1016/j.chom.2015.05.003 26048138

[40] Majlessi L Brosch R . Mycobacterium tuberculosis meets the cytosol: the role of cgas in anti-mycobacterial immunity. Cell Host Microbe. (2015) 17:733–5. doi: 10.1016/j.chom.2015.05.017 26067600

[41] Nandakumar R Tschismarov R Meissner F Prabakaran T Krissanaprasit A Farahani E . Intracellular bacteria engage a sting-tbk1-mvb12b pathway to enable paracrine cgas-sting signalling. Nat Microbiol. (2019) 4:701–13. doi: 10.1038/s41564-019-0367-z 30804548 PMC6433288

[42] Dreier RF Santos JC Broz P . Detecting release of bacterial dsdna into the host cytosol using fluorescence microscopy. Methods Mol Biol (Clifton NJ). (2018) 1714:199–213. doi: 10.1007/978-1-4939-7519-8_13 29177864

[43] Storek KM Gertsvolf NA Ohlson MB Monack DM . Cgas and ifi204 cooperate to produce type I ifns in response to francisella infection. J Immunol (Baltimore Md 1950). (2015) 194:3236–45. doi: 10.4049/jimmunol.1402764 25710914 PMC4367159

[44] Xu L Li M Yang Y Zhang C Xie Z Tang J . Salmonella induces the cgas-sting-dependent type I interferon response in murine macrophages by triggering mtdna release. mBio. (2022) 13:e0363221. doi: 10.1128/mbio.03632-21 35604097 PMC9239183

[45] Waanders L van der Donk LEH Ates LS Maaskant J van Hamme JL Eldering E . Ectopic expression of cgas in salmonella typhimurium enhances sting-mediated ifn-β response in human macrophages and dendritic cells. J Immunother Cancer. (2023) 11(4):e005839. doi: 10.1136/jitc-2022-005839 37072345 PMC10124277

[46] Andrade WA Firon A Schmidt T Hornung V Fitzgerald KA Kurt-Jones EA . Group b streptococcus degrades cyclic-di-amp to modulate sting-dependent type I interferon production. Cell Host Microbe. (2016) 20:49–59. doi: 10.1016/j.chom.2016.06.003 27414497 PMC5382021

[47] Patel S Tucker HR Gogoi H Mansouri S Jin L . Cgas-sting and myd88 pathways synergize in ly6c(hi) monocyte to promote streptococcus pneumoniae-induced late-stage lung ifnγ production. Front Immunol. (2021) 12:699702. doi: 10.3389/fimmu.2021.699702 34512626 PMC8427188

[48] Ruiz-Moreno JS Hamann L Shah JA Verbon A Mockenhaupt FP Puzianowska-Kuznicka M . The common haq sting variant impairs cgas-dependent antibacterial responses and is associated with susceptibility to legionnaires' disease in humans. PloS Pathog. (2018) 14:e1006829. doi: 10.1371/journal.ppat.1006829 29298342 PMC5770077

[49] Yang H Sun P Zhou S Tang Y Li S Li W . Chlamydia psittaci infection induces ifn-I and il-1β through the cgas-sting-irf3/nlrp3 pathway via mitochondrial oxidative stress in human macrophages. Veterinary Microbiol. (2024) 299:110292. doi: 10.1016/j.vetmic.2024.110292 39581075

[50] Sauer JD Sotelo-Troha K von Moltke J Monroe KM Rae CS Brubaker SW . The n-ethyl-n-nitrosourea-induced goldenticket mouse mutant reveals an essential function of sting in the *in vivo* interferon response to listeria monocytogenes and cyclic dinucleotides. Infect Immun. (2011) 79:688–94. doi: 10.1128/iai.00999-10 21098106 PMC3028833

[51] Li N Zhou H Wu H Wu Q Duan M Deng W . Sting-irf3 contributes to lipopolysaccharide-induced cardiac dysfunction, inflammation, apoptosis and pyroptosis by activating nlrp3. Redox Biol. (2019) 24:101215. doi: 10.1016/j.redox.2019.101215 31121492 PMC6529775

[52] Heipertz EL Harper J Walker WE . Sting and Trif contribute to mouse sepsis, depending on severity of the disease model. Shock (Augusta Ga). (2017) 47:621–31. doi: 10.1097/shk.0000000000000771 27755506

[53] Paijo J Döring M Spanier J Grabski E Nooruzzaman M Schmidt T . Cgas senses human cytomegalovirus and induces type I interferon responses in human monocyte-derived cells. PloS Pathog. (2016) 12:e1005546. doi: 10.1371/journal.ppat.1005546 27058035 PMC4825940

[54] Lio CW McDonald B Takahashi M Dhanwani R Sharma N Huang J . Cgas-Sting signaling regulates initial innate control of cytomegalovirus infection. J Virol. (2016) 90:7789–97. doi: 10.1128/jvi.01040-16 27334590 PMC4988162

[55] Zhang G Chan B Samarina N Abere B Weidner-Glunde M Buch A . Cytoplasmic isoforms of Kaposi sarcoma herpesvirus Lana recruit and antagonize the innate immune DNA sensor Cgas. PNAS. (2016) 113:E1034–43. doi: 10.1073/pnas.1516812113 26811480 PMC4776510

[56] Wu JJ Li W Shao Y Avey D Fu B Gillen J . Inhibition of Cgas DNA sensing by a herpesvirus virion protein. Cell Host Microbe. (2015) 18:333–44. doi: 10.1016/j.chom.2015.07.015 26320998 PMC4567405

[57] Ma Z Jacobs SR West JA Stopford C Zhang Z Davis Z . Modulation of the Cgas-Sting DNA sensing pathway by gammaherpesviruses. PNAS. (2015) 112:E4306–15. doi: 10.1073/pnas.1503831112 26199418 PMC4534226

[58] Li XD Wu J Gao D Wang H Sun L Chen ZJ . Pivotal roles of Cgas-Cgamp signaling in antiviral defense and immune adjuvant effects. Sci (New York NY). (2013) 341:1390–4. doi: 10.1126/science.1244040 23989956 PMC3863637

[59] Schoggins JW MacDuff DA Imanaka N Gainey MD Shrestha B Eitson JL . Pan-viral specificity of Ifn-induced genes reveals new roles for Cgas in innate immunity. Nature. (2014) 505:691–5. doi: 10.1038/nature12862 24284630 PMC4077721

[60] Wu J Dobbs N Yang K Yan N . Interferon-independent activities of mammalian Sting mediate antiviral response and tumor immune evasion. Immunity. (2020) 53:115–126.e5. doi: 10.1016/j.immuni.2020.06.009 32640258 PMC7365768

[61] Gao D Wu J Wu YT Du F Aroh C Yan N . Cyclic Gmp-Amp synthase is an innate immune sensor of Hiv and other retroviruses. Sci (New York NY). (2013) 341:903–6. doi: 10.1126/science.1240933 23929945 PMC3860819

[62] Vermeire J Roesch F Sauter D Rua R Hotter D Van Nuffel A . Hiv triggers a Cgas-dependent, Vpu- and Vpr-regulated type I interferon response in Cd4(+) T cells. Cell Rep. (2016) 17:413–24. doi: 10.1016/j.celrep.2016.09.023 27705790

[63] Lahaye X Gentili M Silvin A Conrad C Picard L Jouve M . Nono detects the nuclear Hiv capsid to promote Cgas-mediated innate immune activation. Cell. (2018) 175:488–501.e22. doi: 10.1016/j.cell.2018.08.062 30270045

[64] Holm CK Jensen SB Jakobsen MR Cheshenko N Horan KA Moeller HB . Virus-cell fusion as a trigger of innate immunity dependent on the adaptor Sting. Nat Immunol. (2012) 13:737–43. doi: 10.1038/ni.2350 22706339 PMC3411909

[65] Lusic M Siliciano RF . Nuclear landscape of Hiv-1 infection and integration. Nat Rev Microbiol. (2017) 15:69–82. doi: 10.1038/nrmicro.2016.162 27941817

[66] Zhang Z Zhang C . Regulation of Cgas-Sting signalling and its diversity of cellular outcomes. Nat Rev Immunol. (2025) 25:425–44. doi: 10.1038/s41577-024-01112-7 39774812

[67] An J Durcan L Karr RM Briggs TA Rice GI Teal TH . Expression of cyclic Gmp-Amp synthase in patients with systemic lupus erythematosus. Arthritis Rheumatol (Hoboken NJ). (2017) 69:800–7. doi: 10.1002/art.40002 27863149

[68] Zhou W Richmond-Buccola D Wang Q Kranzusch PJ . Structural basis of human Trex1 DNA degradation and autoimmune disease. Nat Commun. (2022) 13:4277. doi: 10.1038/s41467-022-32055-z 35879334 PMC9314330

[69] Crow YJ Hayward BE Parmar R Robins P Leitch A Ali M . Mutations in the gene encoding the 3'-5' DNA exonuclease Trex1 cause Aicardi-Goutières syndrome at the Ags1 locus. Nat Genet. (2006) 38:917–20. doi: 10.1038/ng1845 16845398

[70] Yi C Li Q Xiao J . Familial chilblain lupus due to a novel mutation in Trex1 associated with Aicardi-Goutie'res syndrome. Pediatr Rheumatol Online J. (2020) 18:32. doi: 10.1186/s12969-020-00423-y 32293470 PMC7158086

[71] Liu Y Jesus AA Marrero B Yang D Ramsey SE Sanchez GAM . Activated Sting in a vascular and pulmonary syndrome. N Engl J Med. (2014) 371:507–18. doi: 10.1056/NEJMoa1312625 25029335 PMC4174543

[72] Decout A Katz JD Venkatraman S Ablasser A . The Cgas-Sting pathway as a therapeutic target in inflammatory diseases. Nat Rev Immunol. (2021) 21:548–69. doi: 10.1038/s41577-021-00524-z 33833439 PMC8029610

[73] Zhang J Zhang L Chen Y Fang X Li B Mo C . The role of Cgas-Sting signaling in pulmonary fibrosis and its therapeutic potential. Front Immunol. (2023) 14:1273248. doi: 10.3389/fimmu.2023.1273248 37965345 PMC10642193

[74] Chen R Du J Zhu H Ling Q . The role of Cgas-Sting signalling in liver diseases. JHEP Reports: Innovation Hepatol. (2021) 3:100324. doi: 10.1016/j.jhepr.2021.100324 34381984 PMC8340306

[75] Li L Liu F Feng C Chen Z Zhang N Mao J . Role of mitochondrial dysfunction in kidney disease: Insights from the Cgas-Sting signaling pathway. Chin Med J. (2024) 137:1044–53. doi: 10.1097/cm9.0000000000003022 38445370 PMC11062705

[76] An C Li Z Chen Y Huang S Yang F Hu Y . The Cgas-Sting pathway in cardiovascular diseases: From basic research to clinical perspectives. Cell Bioscience. (2024) 14:58. doi: 10.1186/s13578-024-01242-4 38720328 PMC11080250

[77] Ke X Hu T Jiang M . Cgas-Sting signaling pathway in gastrointestinal inflammatory disease and cancers. FASEB Journal: Off Publ Fed Am Societies For Exp Biol. (2022) 36:e22029. doi: 10.1096/fj.202101199R 34907606

[78] Yang X Zhao L Pang Y . Cgas-Sting pathway in pathogenesis and treatment of osteoarthritis and rheumatoid arthritis. Front Immunol. (2024) 15:1384372. doi: 10.3389/fimmu.2024.1384372 38765007 PMC11099256

[79] Gulen MF Samson N Keller A Schwabenland M Liu C Glück S . Cgas-Sting drives ageing-related inflammation and neurodegeneration. Nature. (2023) 620:374–80. doi: 10.1038/s41586-023-06373-1 37532932 PMC10412454

[80] Luo J Wang S Yang Q Fu Q Zhu C Li T . Γδ T cell-mediated tumor immunity is tightly regulated by Sting and Tgf-β signaling pathways. Advanced Sci (Weinheim Baden-Wurttemberg Germany). (2025) 12:e2404432. doi: 10.1002/advs.202404432 39573933 PMC11727375

[81] Hopfner KP Hornung V . Molecular mechanisms and cellular functions of Cgas-Sting signalling. Nat Rev Mol Cell Biol. (2020) 21:501–21. doi: 10.1038/s41580-020-0244-x 32424334

[82] Brestoff JR Wilen CB Moley JR Li Y Zou W Malvin NP . Intercellular mitochondria transfer to macrophages regulates white adipose tissue homeostasis and is impaired in obesity. Cell Metab. (2021) 33:270–282.e8. doi: 10.1016/j.cmet.2020.11.008 33278339 PMC7858234

[83] Feng Z Du Z Shu X Zhu L Wu J Gao Q . Role of Rage in obesity-induced adipose tissue inflammation and insulin resistance. Cell Death Discov. (2021) 7:305. doi: 10.1038/s41420-021-00711-w 34686659 PMC8536716

[84] Yang T Qu X Wang X Xu D Sheng M Lin Y . The macrophage Sting-Yap axis controls hepatic steatosis by promoting the autophagic degradation of lipid droplets. Hepatol (Baltimore Md). (2024) 80:1169–83. doi: 10.1097/hep.0000000000000638 37870294 PMC11035483

[85] Yang H Zhao L Kong W Liu S Zhou Q Lang X . Usp18 promotes ferroptosis in lipopolysaccharide-induced human kidney organoids by stabilizing Sting1. Cell Biol Toxicol. (2025) 41:126. doi: 10.1007/s10565-025-10078-8 40833519 PMC12367870

[86] Zhu Z Zhou X Du H Cloer EW Zhang J Mei L . Sting suppresses mitochondrial Vdac2 to govern Rcc growth independent of innate immunity. Advanced Sci (Weinheim Baden-Wurttemberg Germany). (2023) 10:e2203718. doi: 10.1002/advs.202203718 36445063 PMC9875608

[87] Kang J Wu J Liu Q Wu X Zhao Y Ren J . Post-translational modifications of Sting: A potential therapeutic target. Front Immunol. (2022) 13:888147. doi: 10.3389/fimmu.2022.888147 35603197 PMC9120648

[88] Zhang Z Zhou H Ouyang X Dong Y Sarapultsev A Luo S . Multifaceted functions of Sting in human health and disease: From molecular mechanism to targeted strategy. Signal Transduction Targeted Ther. (2022) 7:394. doi: 10.1038/s41392-022-01252-z 36550103 PMC9780328

[89] Kurts C von Vietinghoff S Krebs CF Panzer U . Kidney immunology from pathophysiology to clinical translation. Nat Rev Immunol. (2025) 25:460–76. doi: 10.1038/s41577-025-01131-y 39885266

[90] Costello HM Johnston JG Juffre A Crislip GR Gumz ML . Circadian clocks of the kidney: Function, mechanism, and regulation. Physiol Rev. (2022) 102:1669–701. doi: 10.1152/physrev.00045.2021 35575250 PMC9273266

[91] Birkelo BC Koyner JL Ostermann M Bhatraju PK . The road to precision medicine for acute kidney injury. Crit Care Med. (2024) 52:1127–37. doi: 10.1097/ccm.0000000000006328 38869385 PMC11250999

[92] Garaycoechea JI Quinlan C Luijsterburg MS . Pathological consequences of DNA damage in the kidney. Nat Rev Nephrol. (2023) 19:229–43. doi: 10.1038/s41581-022-00671-z 36702905

[93] Sun C Shi H Zhao X Chang YL Wang X Zhu S . The activation of Cgas-Sting in acute kidney injury. J Inflammation Res. (2023) 16:4461–70. doi: 10.2147/jir.S423232 37842189 PMC10576462

[94] Yuan Q Tang B Zhang C . Signaling pathways of chronic kidney diseases, implications for therapeutics. Signal Transduction Targeted Ther. (2022) 7:182. doi: 10.1038/s41392-022-01036-5 35680856 PMC9184651

[95] Fan MW Tian JL Chen T Zhang C Liu XR Zhao ZJ . Role of cyclic guanosine monophosphate-adenosine monophosphate synthase-stimulator of interferon genes pathway in diabetes and its complications. World J Diabetes. (2024) 15:2041–57. doi: 10.4239/wjd.v15.i10.2041 39493568 PMC11525733

[96] Dhillon P Mulholland KA Hu H Park J Sheng X Abedini A . Increased levels of endogenous retroviruses trigger fibroinflammation and play a role in kidney disease development. Nat Commun. (2023) 14:559. doi: 10.1038/s41467-023-36212-w 36732547 PMC9895454

[97] Bhargava P Schnellmann RG . Mitochondrial energetics in the kidney. Nat Rev Nephrol. (2017) 13:629–46. doi: 10.1038/nrneph.2017.107 28804120 PMC5965678

[98] Basile DP Anderson MD Sutton TA . Pathophysiology of acute kidney injury. Compr Physiol. (2012) 2:1303–53. doi: 10.1002/cphy.c110041 23798302 PMC3919808

[99] Forbes JM . Mitochondria-power players in kidney function? Trends Endocrinol Metabolism: TEM. (2016) 27:441–2. doi: 10.1016/j.tem.2016.05.002 27215468

[100] Li ZL Huang MM Yu MY Nie DF Fu SL Di JJ . Mitochondrial fumarate promotes ischemia/reperfusion-induced tubular injury. Acta Physiologica (Oxford England). (2024) 240:e14121. doi: 10.1111/apha.14121 38409944

[101] Takaori K Nakamura J Yamamoto S Nakata H Sato Y Takase M . Severity and frequency of proximal tubule injury determines renal prognosis. J Am Soc Nephrology: JASN. (2016) 27:2393–406. doi: 10.1681/asn.2015060647 26701981 PMC4978049

[102] Lv LL Feng Y Wen Y Wu WJ Ni HF Li ZL . Exosomal Ccl2 from tubular epithelial cells is critical for albumin-induced tubulointerstitial inflammation. J Am Soc Nephrol JASN. (2018) 29:919–35. doi: 10.1681/asn.2017050523 29295871 PMC5827595

[103] Tang H Yang M Liu Y Liu H Sun L Song P . The Cxcl1-Cxcr2 axis mediates tubular injury in diabetic nephropathy through the regulation of the inflammatory response. Front Physiol. (2021) 12:782677. doi: 10.3389/fphys.2021.782677 34975537 PMC8716832

[104] Chen W Yuan H Cao W Wang T Chen W Yu H . Blocking interleukin-6 trans-signaling protects against renal fibrosis by suppressing Stat3 activation. Theranostics. (2019) 9:3980–91. doi: 10.7150/thno.32352 31281526 PMC6592178

[105] Luo Y Long M Wu X Zeng L . Targeting the Nlrp3 inflammasome in kidney disease: molecular mechanisms, pathogenic roles, and emerging small-molecule therapeutics. Front Immunol. (2025) 16:1703560. doi: 10.3389/fimmu.2025.1703560 41357229 PMC12675237

[106] Zhao ZB Marschner JA Iwakura T Li C Motrapu M Kuang M . Tubular epithelial cell Hmgb1 promotes AKI-CKD transition by sensitizing cycling tubular cells to oxidative stress: a rationale for targeting Hmgb1 during AKI recovery. J Am Soc Nephrol JASN. (2023) 34:394–411. doi: 10.1681/asn.0000000000000024 36857499 PMC10103235

[107] Yamashita N Kusaba T Nakata T Tomita A Ida T Watanabe-Uehara N . Intratubular epithelial-mesenchymal transition and tubular atrophy after kidney injury in mice. Am J Physiol Renal Physiol. (2020) 319:F579–91. doi: 10.1152/ajprenal.00108.2020 32799673

[108] Guerrero-Mauvecin J Villar-Gómez N Rayego-Mateos S Ramos AM Ruiz-Ortega M Ortiz A . Regulated necrosis role in inflammation and repair in acute kidney injury. Front Immunol. (2023) 14:1324996. doi: 10.3389/fimmu.2023.1324996 38077379 PMC10704359

[109] Jin L Yu B Armando I Han F . Mitochondrial DNA-mediated inflammation in acute kidney injury and chronic kidney disease. Oxid Med Cell Longevity. (2021) 2021:9985603. doi: 10.1155/2021/9985603 34306320 PMC8263241

[110] Song Z Xia Y Shi L Zha H Huang J Xiang X . Inhibition of Drp1-Fis1 interaction alleviates aberrant mitochondrial fragmentation and acute kidney injury. Cell Mol Biol Lett. (2024) 29:31. doi: 10.1186/s11658-024-00553-1 38439028 PMC10910703

[111] Shi L Zha H Pan Z Wang J Xia Y Li H . Dusp1 protects against ischemic acute kidney injury through stabilizing mtdna via interaction with Jnk. Cell Death Dis. (2023) 14:724. doi: 10.1038/s41419-023-06247-4 37935658 PMC10630453

[112] Jin L Yu B Wang H Shi L Yang J Wu L . Sting promotes ferroptosis through Ncoa4-dependent ferritinophagy in acute kidney injury. Free Radical Biol Med. (2023) 208:348–60. doi: 10.1016/j.freeradbiomed.2023.08.025 37634745

[113] Zhu H Wang J Miao J Shen M Wang H Huang X . Snord3a regulates Sting transcription to promote ferroptosis in acute kidney injury. Advanced Sci (Weinheim Baden-Wurttemberg Germany). (2024) 11:e2400305. doi: 10.1002/advs.202400305 38962954 PMC11434033

[114] Cao Y Chen X Zhu Z Luo Z Hao Y Yang X . Sting contributes to lipopolysaccharide-induced tubular cell inflammation and pyroptosis by activating endoplasmic reticulum stress in acute kidney injury. Cell Death Dis. (2024) 15:217. doi: 10.1038/s41419-024-06600-1 38485717 PMC10940292

[115] Huang H Han Y Zhang Y Zeng J He X Cheng J . Deletion of pyruvate carboxylase in tubular epithelial cell promotes renal fibrosis by regulating Sqor/Cgas/Sting-mediated glycolysis. Advanced Sci (Weinheim Baden-Wurttemberg Germany). (2025) 12:e2408753. doi: 10.1002/advs.202408753 39836535 PMC11967762

[116] Jiang A Liu J Wang Y Zhang C . Cgas-Sting signaling pathway promotes hypoxia-induced renal fibrosis by regulating Pfkfb3-mediated glycolysis. Free Radical Biol Med. (2023) 208:516–29. doi: 10.1016/j.freeradbiomed.2023.09.011 37714438

[117] Shu S Zhu J Liu Z Tang C Cai J Dong Z . Endoplasmic reticulum stress is activated in post-ischemic kidneys to promote chronic kidney disease. EBioMedicine. (2018) 37:269–80. doi: 10.1016/j.ebiom.2018.10.006 30314894 PMC6286638

[118] Yamada R Yanagita M . When two signals cross paths: Cgas-Sting and ER stress in kidney disease progression. Kidney Int. (2025) 107:227–9. doi: 10.1016/j.kint.2024.11.023 39848744

[119] Andrade-Silva M Dhillon P Sanchez-Navarro A Mukhi D Hu H Kolligundla LP . The critical role of endoplasmic reticulum stress and the stimulator of interferon genes (Sting) pathway in kidney fibrosis. Kidney Int. (2025) 107:302–16. doi: 10.1016/j.kint.2024.10.021 39566842 PMC11757071

[120] He W Mu X Wu X Liu Y Deng J Liu Y . The Cgas-Sting pathway: a therapeutic target in diabetes and its complications. Burns Trauma. (2024) 12:tkad050. doi: 10.1093/burnst/tkad050 38312740 PMC10838060

[121] Feng Z Liao X Peng J Quan J Zhang H Huang Z . Pcsk9 causes inflammation and Cgas/Sting pathway activation in diabetic nephropathy. FASEB J Off Publ Fed Am Societies For Exp Biol. (2023) 37:e23127. doi: 10.1096/fj.202300342RRR 37561547

[122] Zhao QX Yan SB Wang F Li XX Shang GK Zheng ZJ . Sting deficiency alleviates ferroptosis through Fpn1 stabilization in diabetic kidney disease. Biochem Pharmacol. (2024) 222:116102. doi: 10.1016/j.bcp.2024.116102 38428828

[123] Luo X Zhao Y Luo Y Lai J Ji J Huang J . Cytosolic mtdna-Cgas-Sting axis contributes to sepsis-induced acute kidney injury via activating the Nlrp3 inflammasome. Clin Exp Nephrol. (2024) 28:375–90. doi: 10.1007/s10157-023-02448-5 38238499

[124] Yang L Sun L Liu W Rui H Dai H Liu W . Single-cell analysis reveals shared adaptive responses across different types of podocyte injury. Front Immunol. (2025) 16:1698284. doi: 10.3389/fimmu.2025.1698284 41476974 PMC12747916

[125] Yang X Chen Z Luo Z Yang D Hao Y Hu J . Sting deletion alleviates podocyte injury through suppressing inflammation by targeting Nlrp3 in diabetic kidney disease. Cell Signalling. (2023) 109:110777. doi: 10.1016/j.cellsig.2023.110777 37329999

[126] Mitrofanova A Fontanella A Tolerico M Mallela S Molina David J Zuo Y . Activation of stimulator of IFN genes (Sting) causes proteinuria and contributes to glomerular diseases. J Am Soc Nephrol JASN. (2022) 33:2153–73. doi: 10.1681/asn.2021101286 36198430 PMC9731637

[127] Davis SE Khatua AK Popik W . Nucleosomal dsdna stimulates Apol1 expression in human cultured podocytes by activating the Cgas/Ifi16-Sting signaling pathway. Sci Rep. (2019) 9:15485. doi: 10.1038/s41598-019-51998-w 31664093 PMC6820523

[128] Wu J Raman A Coffey NJ Sheng X Wahba J Seasock MJ . The key role of Nlrp3 and Sting in Apol1-associated podocytopathy. J Clin Invest. (2021) 131(20):e136329. doi: 10.1172/jci136329 34651582 PMC8516463

[129] Randle RK Amara VR Popik W . Ifi16 is indispensable for promoting Hif-1α-mediated Apol1 expression in human podocytes under hypoxic conditions. Int J Mol Sci. (2024) 25(6):3324. doi: 10.3390/ijms25063324 38542298 PMC10970439

[130] Vila Cuenca M Hordijk PL Vervloet MG . Most exposed: the endothelium in chronic kidney disease. Nephrology Dialysis Transplant Off Publ Eur Dialysis Transplant Assoc - Eur Renal Assoc. (2020) 35:1478–87. doi: 10.1093/ndt/gfz055 31071222 PMC7473805

[131] Jourde-Chiche N Fakhouri F Dou L Bellien J Burtey S Frimat M . Endothelium structure and function in kidney health and disease. Nat Rev Nephrol. (2019) 15:87–108. doi: 10.1038/s41581-018-0098-z 30607032

[132] Qi H Casalena G Shi S Yu L Ebefors K Sun Y . Glomerular endothelial mitochondrial dysfunction is essential and characteristic of diabetic kidney disease susceptibility. Diabetes. (2017) 66:763–78. doi: 10.2337/db16-0695 27899487 PMC5319717

[133] Casalena GA Yu L Gil R Rodriguez S Sosa S Janssen W . The diabetic microenvironment causes mitochondrial oxidative stress in glomerular endothelial cells and pathological crosstalk with podocytes. Cell Communication Signaling CCS. (2020) 18:105. doi: 10.1186/s12964-020-00605-x 32641054 PMC7341607

[134] Daehn I Casalena G Zhang T Shi S Fenninger F Barasch N . Endothelial mitochondrial oxidative stress determines podocyte depletion in segmental glomerulosclerosis. J Clin Invest. (2014) 124:1608–21. doi: 10.1172/jci71195 24590287 PMC3973074

[135] Wu J Ma Z Raman A Beckerman P Dhillon P Mukhi D . Apol1 risk variants in individuals of African genetic ancestry drive endothelial cell defects that exacerbate sepsis. Immunity. (2021) 54:2632–2649.e6. doi: 10.1016/j.immuni.2021.10.004 34715018 PMC9338439

[136] Boyle SC Liu Z Kopan R . Notch signaling is required for the formation of mesangial cells from a stromal mesenchyme precursor during kidney development. Dev (Cambridge England). (2014) 141:346–54. doi: 10.1242/dev.100271 24353058 PMC4074211

[137] Wu J Shao X Shen J Lin Q Zhu X Li S . Downregulation of Pparα mediates Fabp1 expression, contributing to Iga nephropathy by stimulating ferroptosis in human mesangial cells. Int J Biol Sci. (2022) 18:5438–58. doi: 10.7150/ijbs.74675 36147466 PMC9461665

[138] Li L Fu H Liu Y . The fibrogenic niche in kidney fibrosis: components and mechanisms. Nat Rev Nephrol. (2022) 18:545–57. doi: 10.1038/s41581-022-00590-z 35788561

[139] Ban JQ Ao LH He X Zhao H Li J . Advances in macrophage-myofibroblast transformation in fibrotic diseases. Front Immunol. (2024) 15:1461919. doi: 10.3389/fimmu.2024.1461919 39445007 PMC11496091

[140] Jiao B An C Du H Tran M Yang D Zhao Y . Genetic deficiency or pharmacological inhibition of Cgas-Sting signalling suppresses kidney inflammation and fibrosis. Br J Pharmacol. (2025) 182:1741–62. doi: 10.1111/bph.17412 39833988

[141] Zeng H Gao Y Yu W Liu J Zhong C Su X . Pharmacological inhibition of Sting/Tbk1 signaling attenuates myeloid fibroblast activation and macrophage to myofibroblast transition in renal fibrosis. Front Pharmacol. (2022) 13:940716. doi: 10.3389/fphar.2022.940716 35924048 PMC9340478

[142] Sheridan C . Drug developers switch gears to inhibit Sting. Nat Biotechnol. (2019) 37:199–201. doi: 10.1038/s41587-019-0060-z 30833772

[143] Haag SM Gulen MF Reymond L Gibelin A Abrami L Decout A . Targeting Sting with covalent small-molecule inhibitors. Nature. (2018) 559:269–73. doi: 10.1038/s41586-018-0287-8 29973723

[144] Zhou S Cheng F Zhang Y Su T Zhu G . Engineering and delivery of Cgas-Sting immunomodulators for the immunotherapy of cancer and autoimmune diseases. Acc Chem Res. (2023) 56:2933–43. doi: 10.1021/acs.accounts.3c00394 37802125 PMC10882213

[145] Dai J Huang YJ He X Zhao M Wang X Liu ZS . Acetylation blocks Cgas activity and inhibits self-DNA-induced autoimmunity. Cell. (2019) 176:1447–1460.e14. doi: 10.1016/j.cell.2019.01.016 30799039 PMC8274936

[146] Vincent J Adura C Gao P Luz A Lama L Asano Y . Small molecule inhibition of Cgas reduces interferon expression in primary macrophages from autoimmune mice. Nat Commun. (2017) 8:750. doi: 10.1038/s41467-017-00833-9 28963528 PMC5622107

[147] Hong Z Mei J Li C Bai G Maimaiti M Hu H . Sting inhibitors target the cyclic dinucleotide binding pocket. PNAS. (2021) 18(24):e2105465118. doi: 10.1073/pnas.2105465118 34099558 PMC8214703

[148] Gong W Lu L Zhou Y Liu J Ma H Fu L . The novel Sting antagonist H151 ameliorates cisplatin-induced acute kidney injury and mitochondrial dysfunction. Am J Physiol Renal Physiol. (2021) 320:F608–16. doi: 10.1152/ajprenal.00554.2020 33615891

[149] Xia L Jiang JH Liu JY Zhang TY Dong YX Liu QH . H-151 attenuates lipopolysaccharide-induced acute kidney injury by inhibiting the Sting-Tbk1 pathway. Renal Failure. (2024) 46:2363591. doi: 10.1080/0886022x.2024.2363591 38856314 PMC11168233

[150] Leng X Li Q Chen W Feng H Li L Yu L . C-176 inhibits macrophage polarization towards M1-subtype and ameliorates LPS induced acute kidney injury. Eur J Pharmacol. (2024) 984:177028. doi: 10.1016/j.ejphar.2024.177028 39366502

[151] Zhi H Fu H Zhang Y Fan N Zhao C Li Y . Progress of Cgas-Sting signaling pathway-based modulation of immune response by traditional Chinese medicine in clinical diseases. Front Immunol. (2024) 15:1510628. doi: 10.3389/fimmu.2024.1510628 39737190 PMC11683013

[152] Fu G Zhao Y Mao C Liu Y . Enhancing nano-immunotherapy of cancer through cgas-sting pathway modulation. Biomater Sci. (2025) 13:2235–60. doi: 10.1039/d4bm01532k 40111213

[153] Liang L Zeng J Liu R Zheng Z Lyu D Zhang X . Polydatin attenuates diabetic renal inflammatory fibrosis via the inhibition of sting pathway. Biochem Pharmacol. (2024) 226:116373. doi: 10.1016/j.bcp.2024.116373 38885772

[154] Zhao M Peng N Zhou Y Qu Y Cao M Zou Q . The immunoregulatory effects of total glucosides of peony in autoimmune diseases. J Leukocyte Biol. (2025) 117(2):qiae095. doi: 10.1093/jleuko/qiae095 38626175

[155] Xiu Y Wang S Zhang P Li C Wu Z Wen J . Total glucosides of paeony alleviates cgas-sting-mediated diseases by blocking the sting-irf3 interaction. Chin J Natural Medicines. (2024) 22:402–15. doi: 10.1016/s1875-5364(24)60572-8 38796214

[156] D'Amico D Andreux PA Valdés P Singh A Rinsch C Auwerx J . Impact of the natural compound urolithin a on health, disease, and aging. Trends Mol Med. (2021) 27:687–99. doi: 10.1016/j.molmed.2021.04.009 34030963

[157] Zhang C Song Y Chen L Chen P Yuan M Meng Y . Urolithin a attenuates hyperuricemic nephropathy in fructose-fed mice by impairing sting-nlrp3 axis-mediated inflammatory response via restoration of parkin-dependent mitophagy. Front Pharmacol. (2022) 13:907209. doi: 10.3389/fphar.2022.907209 35784701 PMC9240289

[158] Zheng Y Ren W Zhang L Zhang Y Liu D Liu Y . A review of the pharmacological action of astragalus polysaccharide. Front Pharmacol. (2020) 11:349. doi: 10.3389/fphar.2020.00349 32265719 PMC7105737

[159] Sun C Zhao X Wang X Yu Y Shi H Tang J . Astragalus polysaccharide mitigates rhabdomyolysis-induced acute kidney injury via inhibition of m1 macrophage polarization and the cgas-sting pathway. J Inflammation Res. (2024) 17:11505–27. doi: 10.2147/jir.S494819 39735897 PMC11675321

[160] Ma Y Bai B Liu D Shi R Zhou Q . Shenqi fuzheng injection reduces cisplatin-induced kidney injury via cgas/sting signaling pathway in breast cancer mice model. Breast Cancer (Dove Med Press). (2024) 16:451–69. doi: 10.2147/bctt.S475860 39165276 PMC11335009

[161] Peng Y Wu S Xu Y Ye X Huang X Gao L . Huangqi-danshen decoction alleviates renal fibrosis through targeting scd1 to modulate cgas/sting signaling. J Ethnopharmacol. (2025) 342:119364. doi: 10.1016/j.jep.2025.119364 39832629

[162] Shi W Xu G Gao Y Yang H Liu T Zhao J . Compound danshen dripping pill effectively alleviates cgas-sting-triggered diseases by disrupting sting-tbk1 interaction. Phytomedicine: Int J Phytotherapy Phytopharmacology. (2024) 128:155404. doi: 10.1016/j.phymed.2024.155404 38507852

[163] Zheng M Hu Z Wang Y Wang C Zhong C Cui W . Zhen wu decoction represses renal fibrosis by invigorating tubular nrf2 and tfam to fuel mitochondrial bioenergetics. Phytomedicine: Int J Phytotherapy Phytopharmacology. (2023) 108:154495. doi: 10.1016/j.phymed.2022.154495 36257219

[164] Liu S Gao X Wang Y Wang J Qi X Dong K . Baicalein-loaded silk fibroin peptide nanofibers protect against cisplatin-induced acute kidney injury: fabrication, characterization and mechanism. Int J Pharm. (2022) 626:122161. doi: 10.1016/j.ijpharm.2022.122161 36058409

[165] Qi X Wang J Fei F Gao X Wu X Shi D . Myricetin-loaded nanomicelles protect against cisplatin-induced acute kidney injury by inhibiting the dna damage-cgas-sting signaling pathway. Mol Pharmaceutics. (2023) 20:136–46. doi: 10.1021/acs.molpharmaceut.2c00520 36326450

[166] Gao X Wang J Wang Y Liu S Dong K Wu J . Fucoidan-ferulic acid nanoparticles alleviate cisplatin-induced acute kidney injury by inhibiting the cgas-sting pathway. Int J Biol Macromol. (2022) 223:1083–93. doi: 10.1016/j.ijbiomac.2022.11.062 36372101

[167] Liu S Gao X Yin Y Wang J Dong K Shi D . Silk fibroin peptide self-assembled nanofibers delivered naringenin to alleviate cisplatin-induced acute kidney injury by inhibiting mtdna-cgas-sting pathway. Food Chem Toxicology: Int J Published For Br Ind Biol Res Assoc. (2023) 177:113844. doi: 10.1016/j.fct.2023.113844 37244599

[168] Jiang T Zhu F Gao X Wu X Zhu W Guo C . Naringenin loaded fucoidan/polyvinylpyrrolidone nanoparticles protect against folic acid induced acute kidney injury *in vitro* and *in vivo*. Colloids Surfaces B Biointerfaces. (2025) 245:114343. doi: 10.1016/j.colsurfb.2024.114343 39486374

[169] Gao X Yin Y Liu S Dong K Wang J Guo C . Fucoidan-proanthocyanidins nanoparticles protect against cisplatin-induced acute kidney injury by activating mitophagy and inhibiting mtdna-cgas/sting signaling pathway. Int J Biol Macromol. (2023) 245:125541. doi: 10.1016/j.ijbiomac.2023.125541 37355076

[170] Chen Q Yang Y Ying X Huang C Chen J Wang J . Hierarchical targeting nanodrug with holistic dna protection for effective treatment of acute kidney injury. Advanced Sci (Weinheim Baden-Wurttemberg Germany). (2025) 12:e2411254. doi: 10.1002/advs.202411254 39703158 PMC11809360

[171] Li Q Wu P Du Q Hanif U Hu H Li K . Cgas-sting, an important signaling pathway in diseases and their therapy. MedComm. (2024) 5:e511. doi: 10.1002/mco2.511 38525112 PMC10960729

[172] Elmetwalli A . Ferroptosis and the cgas-sting pathway into precision nano-immuno-theranostics: a mechanistic paradigm for reversing drug resistance in hepatocellular carcinoma. Drug Resistance Updates: Rev Commentaries Antimicrobial Anticancer Chemotherapy. (2026) 84:101326. doi: 10.1016/j.drup.2025.101326 41275854

